# ZZW-115–dependent inhibition of NUPR1 nuclear translocation sensitizes cancer cells to genotoxic agents

**DOI:** 10.1172/jci.insight.138117

**Published:** 2020-09-17

**Authors:** Wenjun Lan, Patricia Santofimia-Castaño, Mirna Swayden, Yi Xia, Zhengwei Zhou, Stephane Audebert, Luc Camoin, Can Huang, Ling Peng, Ana Jiménez-Alesanco, Adrián Velázquez-Campoy, Olga Abián, Gwen Lomberk, Raul Urrutia, Bruno Rizzuti, Vincent Geli, Philippe Soubeyran, José L. Neira, Juan Iovanna

**Affiliations:** 1 Centre de Recherche en Cancérologie de Marseille (CRCM), INSERM U1068, CNRS UMR 7258, Aix-Marseille Université and Institut Paoli-Calmettes, Parc Scientifique et Technologique de Luminy, Equipe Labélisée Ligue Nationale Contre le Cancer, Marseille, France.; 2 Aix-Marseille Université, CNRS, Centre Interdisciplinaire de Nanoscience de Marseille, UMR 7325, Equipe Labellisée Ligue Contre le Cancer, Parc Scientifique et Technologique de Luminy, Marseille, France.; 3 Chongqing Key Laboratory of Natural Product Synthesis and Drug Research, School of Pharmaceutical Sciences, Chongqing University, No.55 Daxuecheng South Road, Chongqing, China.; 4 Instituto de Biocomputación y Física de Sistemas Complejos (BIFI), Joint Units IQFR-CSIC-BIFI, and GBsC-CSIC-BIFI, Universidad de Zaragoza, Spain.; 5 Instituto de Investigación Sanitaria de Aragón (IIS Aragon), Zaragoza, Spain.; 6 Centro de Investigación Biomédica en Red en el Área Temática de Enfermedades Hepáticas y Digestivas (CIBERehd), Madrid, Spain.; 7 Departamento de Bioquímica y Biología Molecular y Celular, Universidad de Zaragoza, Zaragoza, Spain.; 8 Fundacion ARAID, Gobierno de Aragón, Zaragoza, Spain.; 9 Instituto Aragonés de Ciencias de la Salud (IACS), Zaragoza, Spain.; 10 Division of Research, Department of Surgery and the Genomic Sciences and Precision Medicine Center (GSPMC), Medical College of Wisconsin, Milwaukee, Wisconsin, USA.; 11 CNR-NANOTEC, Licryl-UOS Cosenza and CEMIF.Cal, Department of Physics, University of Calabria, Arcavacata di Rende, Cosenza, Italy.; 12 Instituto de Biología Molecular y Celular, Universidad Miguel Hernández, Edificio Torregaitán, Alicante, Spain.

**Keywords:** Cell Biology, Oncology, Cancer, Cell stress

## Abstract

Establishing the interactome of the cancer-associated stress protein Nuclear Protein 1 (NUPR1), we found that it binds to several hundreds of proteins, including proteins involved in nuclear translocation, DNA repair, and key factors of the SUMO pathway. We demonstrated that the NUPR1 inhibitor ZZW-115, an organic synthetic molecule, competes with importins for the binding to the NLS region of NUPR1, thereby inhibiting its nuclear translocation. We hypothesized, and then proved, that inhibition of NUPR1 by ZZW-115 sensitizes cancer cells to DNA damage induced by several genotoxic agents. Strikingly, we found that treatment with ZZW-115 reduced SUMOylation of several proteins involved in DNA damage response (DDR). We further report that the presence of recombinant NUPR1 improved the SUMOylation in a cell-free system, indicating that NUPR1 directly stimulates the SUMOylation machinery. We propose that ZZW-115 sensitizes cancer cells to genotoxic agents by inhibiting the nuclear translocation of NUPR1 and thereby decreasing the SUMOylation-dependent functions of key proteins involved in the DDR.

## Abstract 2

The ZZW-115-dependent inhibition of NUPR1 nuclear translocation sensitizes cancer cells to genotoxic agents by affecting SUMOylation.

## Graphical abstract

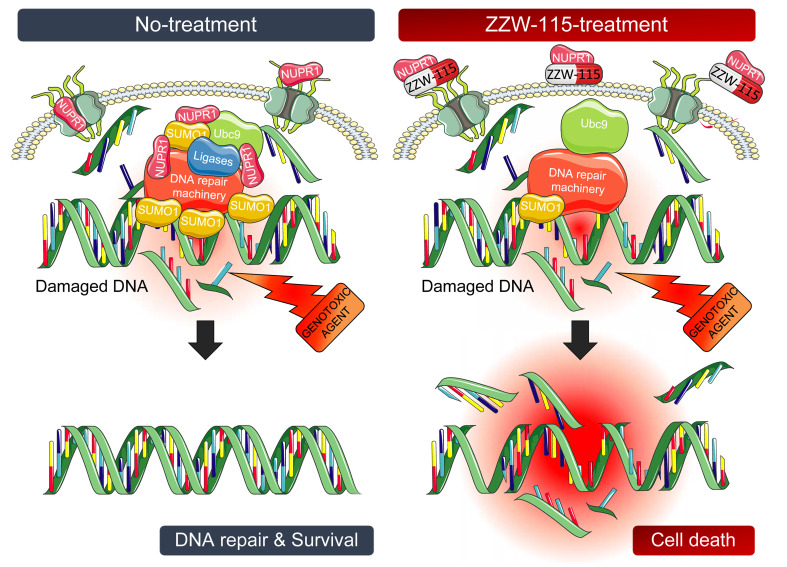

## Introduction

Nuclear Protein 1 (NUPR1) is a nuclear intrinsically disordered protein (IDP) of 82 amino acids that plays an important role in pancreatic ductal adenocarcinoma (PDAC) (1, 2), as well as other cancers in which its genetic inactivation induces tumor growth arrest (3–9). Drug development targeting IDPs is challenging due to their dynamic structural features for which a conventional structure-based drug design is not feasible. We recently developed an efficient multidisciplinary strategy by combining biophysical, biochemical, bioinformatics, and biological approaches to perform molecular screening for selecting potential drug candidates targeting NUPR1 (10). For fulfilling this aim, we have employed a screening method based on measuring fluorescence thermal denaturation and identified the well-known antipsychotic agent trifluoperazine (TFP) as a ligand for NUPR1. Although a strong antitumoral effect of TFP has been shown in in vitro as well as in vivo studies (10), this molecule had a strong narcoleptic side effect at the therapeutic doses. For this reason, a family of TFP-derived compounds was synthesized based on in silico screening (11). Among these molecules, ZZW-115 was the most efficient, as it displayed the best affinity for NUPR1 in vitro and showed antitumoral activity 10 times higher than TFP when tested on a large panel of primary PDAC-derived cells and several nonpancreatic cancer cells (12). In addition, ZZW-115 showed a dose-dependent tumor regression in xenografted mice, leading to a nearly total disappearance of tumors after 30 days of treatment in 5 independent PDAC models, including an immunocompetent mice model. In all cases, no apparent neurological effect was observed when ZZW-115 was used. Since targeting NUPR1 by ZZW-115 is highly efficient for treating cancers, it became essential to determine the molecular mechanisms by which ZZW-115 exerts its antitumoral activity and eventually to determine other anticancer-associated functions.

Active transport from the cytoplasm to nucleus occurs via a family of nuclear transport receptors known as importins (or karyopherins), together with other proteins including nucleoporins (NUPs) (13). The classical importin pathway is initiated by recognition of proteins having a nuclear localization sequence (NLS) by importin-α. The resulting complex is transported through the pore in the nucleus by importin-β and, thus, forms a larger complex, which interacts with the FG-rich regions belonging to NUP proteins (14).

Here, we used NUPR1 immunoprecipitation followed by mass spectrometry analysis to generate the NUPR1 interactome. We identified 656 NUPR1 interacting partners, among which nuclear proteins were highly abundant. We demonstrated that ZZW-115 inhibited the NUPR1 function by hampering its nuclear translocation by interfering with its recognition by NUPs. We also found that NUPR1 interacted with 54 proteins involved in DNA repair machinery, suggesting that NUPR1 could participate in this process, as it was previously suggested in studies with male specific lethal protein 1 (MSL1) (15). This led us to hypothesize that administration of ZZW-115 could improve the effect of the genotoxic agents by hampering the DNA damage response (DDR) and DNA repair processes. This hypothesis was confirmed both in vitro and in vivo and was explained by interference with the SUMOylation of key DDR proteins, including several NUPR1 partners, such as TP53, MRE11, and KDM1A. Finally, using a complete cell-free system, we demonstrated that NUPR1 improved the SUMOylation of partner proteins, a function also inhibited by ZZW-115, indicating that NUPR1 may affect the SUMOylation — and therefore activation — of numerous molecules involved in DDR. All these processes are hampered by the presence of ZZW-115, as NUPR1 could not be translocated into the nucleus and favor the SUMOylation of key DDR actors.

## Results

### Identification of Flag-NUPR1 partners.

To identify the partners of NUPR1, lysate of MiaPaCa-2 cells expressing a Flag-tagged NUPR1 fusion protein was used. Then, an immunoprecipitation was performed with anti-Flag antibodies conjugated beads followed by Flag peptide elution and a liquid-chromatography tandem mass spectrometry (LC-MS/MS) proteomic analysis of the trypsin digested precipitated proteins. We identified 656 proteins capable of interacting with NUPR1. As expected, since NUPR1 is mainly nuclear, the majority of the partners were nuclear proteins (Supplemental Table 1; supplemental material available online with this article; https://doi.org/10.1172/jci.insight.138117DS1). A bioinformatics analysis using the STRING protein-protein interaction database showed a significant enrichment of proteins involved in the following processes: nucleocytoplasmic transport, DNA repair, cellular response to DNA damage stimulus, RNA processing, RNA splicing, SUMO E3 ligases with their SUMOylated target proteins, and SUMOylation of proteins devoted to DDR and repair (Table 1). These results indicate that NUPR1 could play a significant role in all those cellular processes. In all likelihood, some of these proteins interact directly to NUPR1, whereas some other are coprecipitated because they are part of multiprotein complexes. A more detailed study will be further performed to determine the direct partners of NUPR1.

### ZZW-115 inhibits the nuclear transport of NUPR1 by competing with importins.

**Figure 1:**
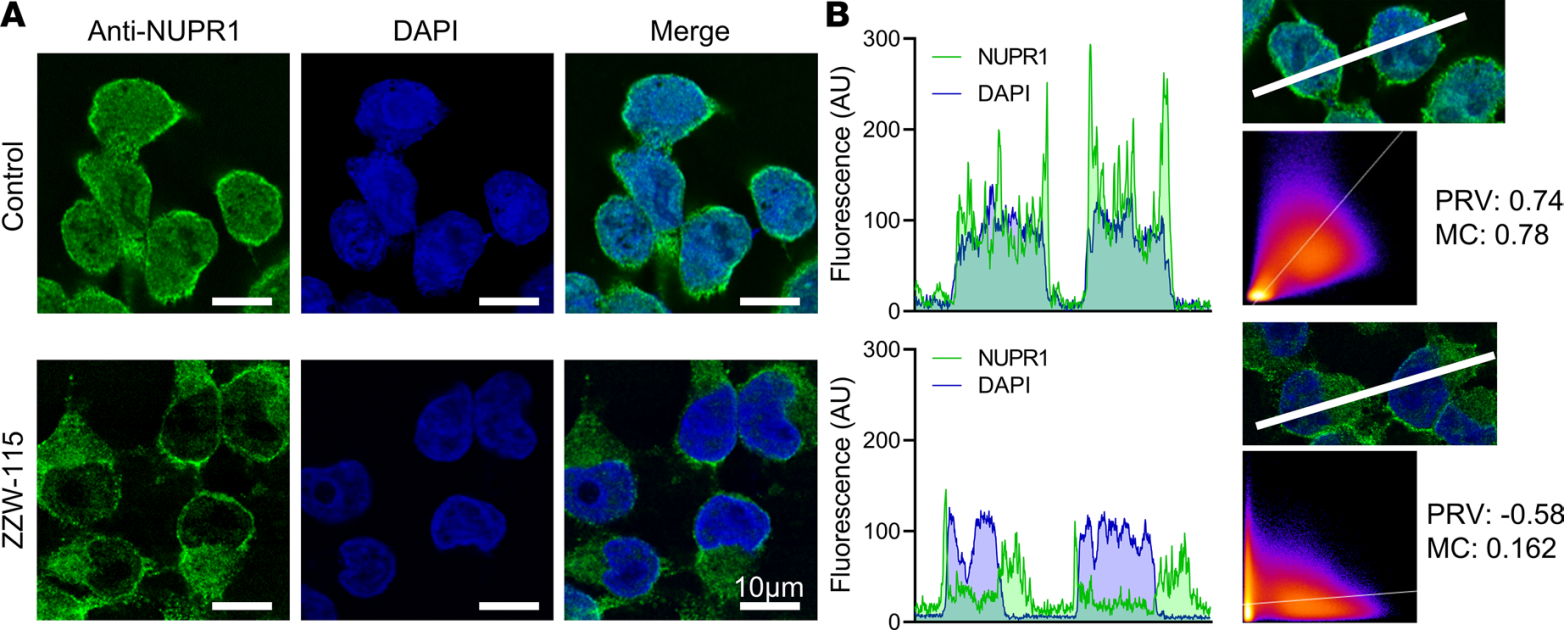
ZZW-115 inhibited NUPR1 nuclear translocation. (**A**) MiaPaCa-2 cells were treated with ZZW-115 (5 μM) for 6 hours. Immunofluorescence with rabbit anti-NUPR1 primary antibody and Alexa 488–labeled goat anti-rabbit secondary antibody were used to reveal the localization of the protein. DAPI staining was used to detect cell nuclei, and it was combined with the Alexa 488 fluorescence in the merged panel. Scale bars: 10 µm. representative experiment is shown (*n* = 3). (**B**) Intensity profiles along the white line in the image are shown. Colocalization scatter plot, Pearson’s R value (PRV), and Mander’s coefficient (MC) were calculated by using the ImageJ Coloc2 plugin; a representative experiment is shown (*n* = 3).

NUPR1 contains a canonical bipartite domain of positively charged amino acids, typical of NLS, localized from residues 63–78 (16). The interactome of NUPR1 revealed that this protein was bound to 30 components of nuclear pore including several importins or karyopherins (KPNA1, KPNA2, KPNA3, KPNA4, and KPNA6) and 17 NUPs. In our previous work, we have shown that NUPR1 binds ZZW-115 via residues around Ala33 and Thr68 (12). Because Thr68 belongs to the NLS region of NUPR1, it is reasonable that ZZW-115 can hinder the interaction between NUPR1 (through its NLS) and importins, and then block the NUPR1 nuclear translocation. Therefore, by using NUPR1 immunofluorescence staining, we studied the potential impact of ZZW-115 on the intracellular location of NUPR1. We found that treatment with ZZW-115 inhibited almost completely the translocation of NUPR1 from the cytoplasm to the nucleus from 78% in control cells to 16% in ZZW-115 treated cells (Figure 1, A and B). We verified this observation in vivo by treating nude mice with xenografts, generated by injecting MiaPaCa-2 cells, with ZZW-115 (5 mg/kg/day for 30 days). The subcellular localization of NUPR1 was studied by confocal immunofluorescence. As expected, NUPR1 showed nuclear localization in untreated xenografts; however, in treated animals, the nuclear localization was strongly decreased as presented in Supplemental Figure 1, A and B. An interesting point to be noted is that the total fluorescence in treated tumors was strongly decreased. Taken together, these results led us to the conclusion that ZZW-115 inactivated NUPR1 by preventing its translocation into the nucleus, where it is expected to play its essential roles regarding cell survival, especially under stress conditions.

### NUPR1 and importin-α3 interact in vitro and in cellulo.

**Figure 2:**
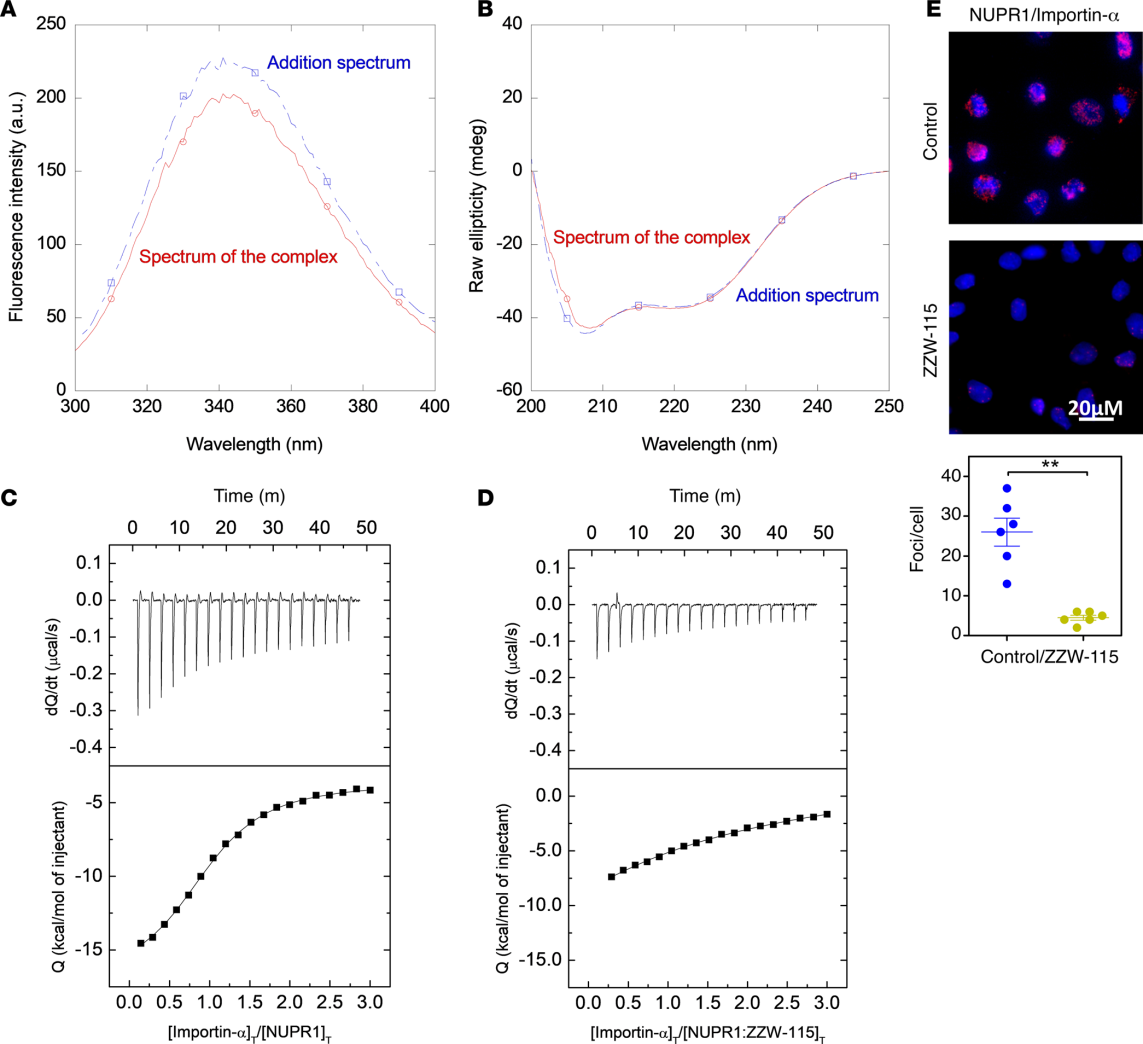
NUPR1 interacted with importin-α3 in vitro. (**A**) Fluorescence spectrum of the complex formed by importin-α3 and NUPR1 (red) and that obtained by the addition of the spectra of both isolated biomolecules after excitation at 280 nm (blue). (**B**) Far-UV CD spectrum of the complex formed by importin-α3 and NUPR1 (red) and that obtained by the addition of the spectra of both isolated biomolecules (blue). (**C** and **D**) ITC raw data (top, thermal power [dQ/dt], as a function of time [t]) and titration curve or binding isotherm (bottom, ligand-normalized injection heats [Q], as a function of the reactants molar ratio) for the interaction between importin-α3 and NUPR1 in the absence (**C**) or presence (**D**) of ZZW-115. (**E**) PLA was performed in MiaPaCa-2 cells transfected with a plasmid expressing importin-α3–Flag in the presence or absence of ZZW-115 (5 μM) for 6 hours. Mouse anti-Flag and rabbit anti-NUPR1 antibodies were used. A representative experiment is shown (*n* = 3). ImageJ was used to count the number of red dots. Data represent mean ± SEM of 6 field, Student’s 2-tailed unpaired *t* test was used, **P < 0.01.

Since we had observed an interaction between importins and NUPR1 in its interactome, we decided to investigate the interaction between NUPR1 and importin-α3 (KPNA4) in vitro by using fluorescence and circular dichroism (CD). We observed changes in the fluorescence spectra after excitation at either 280 or 295 nm; since NUPR1 has only 2 tyrosines (Tyr30 and Tyr36), the changes observed in the fluorescence spectrum by excitation at 295 nm must be due to changes in the environment around at least 1 of the 6 tryptophans in importin-α3 (Figure 2A). Conversely, the far-UV CD spectra did not show any change, suggesting that the secondary structure of importin-α3 did not change upon binding (Figure 2B). Furthermore, the CD results suggest that NUPR1 remained disordered upon binding (as it happens in other complexes formed by the protein) (15, 17). To further demonstrate that there was binding between NUPR1 and importin-α3 in vitro, we provide a quantitative measurement for this interaction. We carried out isothermal titration calorimetry (ITC) experiments in the absence and in the presence of ZZW-115. The results (Figure 2C and Table 2) indicate that: (a) the affinity of NUPR1 for importin-α3 (association constant of 6.9 × 10^5^ M^–1^, and dissociation constant of 1.4 μM) was similar to that shown by NUPR1 toward other biomolecules (15, 17, 18) and for ZZW-115 (association constant of 4.7 × 10^5^ M^–1^ and dissociation constant of 2.1 μM; ref. 12); and (b) in the presence of ZZW-115, a 25-fold reduction in the affinity between NUPR1 and importin-α3 was observed (Figure 2D). The 25-fold reduction caused by ZZW-115 at a concentration of 100 μM obtained with the binary system approximation corresponds to a heterotropic cooperativity constant equal to 0.02, which is in good agreement considering the experimental error with the value of 0.03 obtained with the analysis performed by solving the exact ternary equilibrium. Alternatively, a 21-fold reduction in the affinity for NUPR1 interacting with importin-α3 caused by the presence of ZZW-115 at 100 μM was calculated from the ternary equilibrium analysis, in agreement within the experimental error, with the 25-fold reduction obtained from the binary system approximation. If ZZW-115 was a purely competitive inhibitor, a 45-fold reduction in the affinity for NUPR1 interacting with importin-α3 would be elicited by the presence of ZZW-115 at 100 μM, suggesting that mixed inhibition is possible and the formation of the (nonproductive) ternary complex NUPR1/ZZW-115/importin-α3 cannot be ruled out. Then, we confirmed this interaction using the proximity ligation assay (PLA) in MiaPaCa-2 cells transfected with a plasmid expressing the importin-α3–Flag. Figure 2E shows that NUPR1 and importin-α3–Flag interact, and this interaction is strongly diminished by the treatment with ZZW-115. Therefore, we have quantitatively shown that there was binding in vitro and in cellulo between NUPR1 and importin-α3, and the presence of ZZW-115 hampered that interaction.

### Treatment with ZZW-115 sensitizes cancer cells to genotoxic-induced DNA damage.

**Figure 3:**
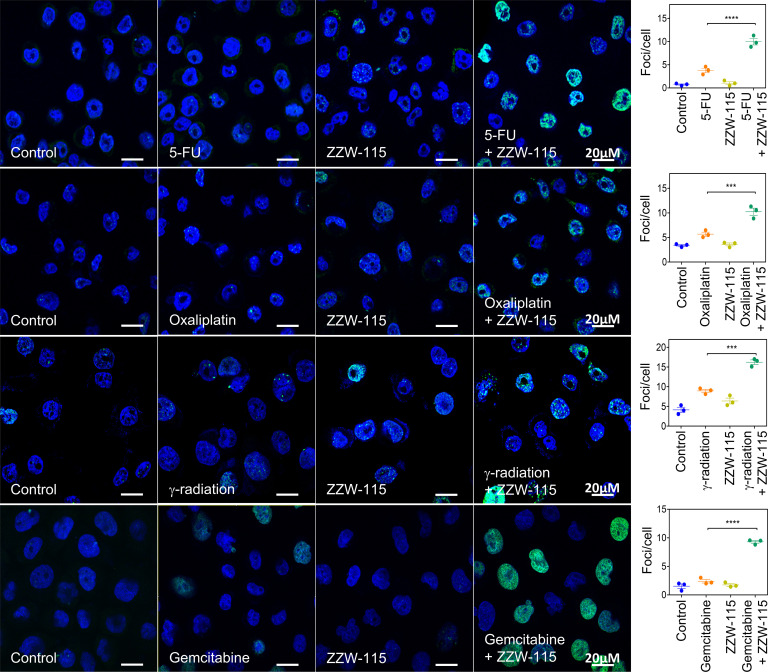
NUPR1 inhibition by ZZW-115 potentiated the efficacy of genotoxic agents in primary PDAC cells. The efficacy of different genotoxic agents (5-FU, Oxaliplatin, γ-radiation, and gemcitabine) to generate DNA breaks in AOIPC primary PDAC cells and the boosting effect of ZZW-115 was evaluated by γH2AX immunofluorescence staining. Quantifications of 3 independent experiments were used to evaluate the statistical significance, and they are shown as graphics. ***P < 0.001; ****P < 0.0001 (1-way ANOVA, Tukey’s post hoc test). Data represent mean ± SEM, *n* = 3.

The NUPR1 interactome revealed strong and specific abundance of proteins involved in DNA repair processes (54 NUPR1 interactors out of 491 proteins in this category) and in cellular response to DNA damage stimulus processes (63 NUPR1 interactors out of 749 proteins in this category) (Table 1). Proteins of these functional complexes, which interact directly with NUPR1, remain to be defined. Hence, we hypothesized that NUPR1 could be involved in the DNA repair process and that, by blocking this particular function of NUPR1, ZZW-115 could be used to improve the efficacy of genotoxic agents. We have had preliminary evidence that NUPR1 could be involved in DNA repair by using several spectroscopic and biophysical techniques because of its interaction with MSL1 (15). Several cancer cell types (PDAC, AOIPC and MiaPaCa-2; glioblastoma, U87 and U251; colon cancer, HT29) were treated with different genotoxic agents such as 5-fluorouracile (5-FU), oxaliplatin, gemcitabine, temozolomide (TMZ), or γ-radiation, alone or in combination with ZZW-115. Then, DNA damage was quantified by counting the number of γH2AX foci (Figure 3 and Supplemental Figure 2). As expected, DNA lesions caused by all genotoxic agents were strongly increased when cells were cotreated with ZZW-115. Importantly, this potentiation effect was genotoxic agent independent and cell type independent (Figure 3 and Supplemental Figure 2). Remarkably, when nontransformed cancer-associated fibroblasts (CAF) were treated with ZZW-115, no improvement of DNA damage induced by 5-FU was observed (Supplemental Figure 2). As expected, no effect of ZZW-115 was observed when cells were treated with a nongenotoxic agent like sorafenib, a multikinase inhibitor (Supplemental Figure 2). In addition, to demonstrate that the phenotype observed upon ZZW-115 treatment is due to NUPR1’s inhibition, MiaPaCa-2 cells were transfected with siRNAs against NUPR1. Interestingly, NUPR1-depleted cells were more sensitive to 5-FU alone than control cells. However, while — in the siRNA control cells — ZZW-115 induced a synergic effect in combination with 5-FU, in the NUPR1-depleted cells, ZZW-115 did not increase the levels of DNA-damage (Supplemental Figure 2). Thus, we concluded that NUPR1 is involved in the DDR and that ZZW-115 targeting of NUPR1 sensitizes cancer cells to any genotoxic treatment.

### Cotreatments with ZZW-115 improve the efficacy of genotoxic agents on PDAC and glioblastoma in vivo.

Because the compound ZZW-115 was capable of improving the DNA damage induced by several genotoxic agents on different cell types in vitro, we wondered whether this could also happen in vivo. To this purpose, we chose to follow the development of MiaPaCa-2 PDAC and U87 glioblastoma cells xenografted in mice treated with 5-FU and/or ZZW-115 or TMZ and/or ZZW-115, respectively.

### PDAC.

**Figure 4:**
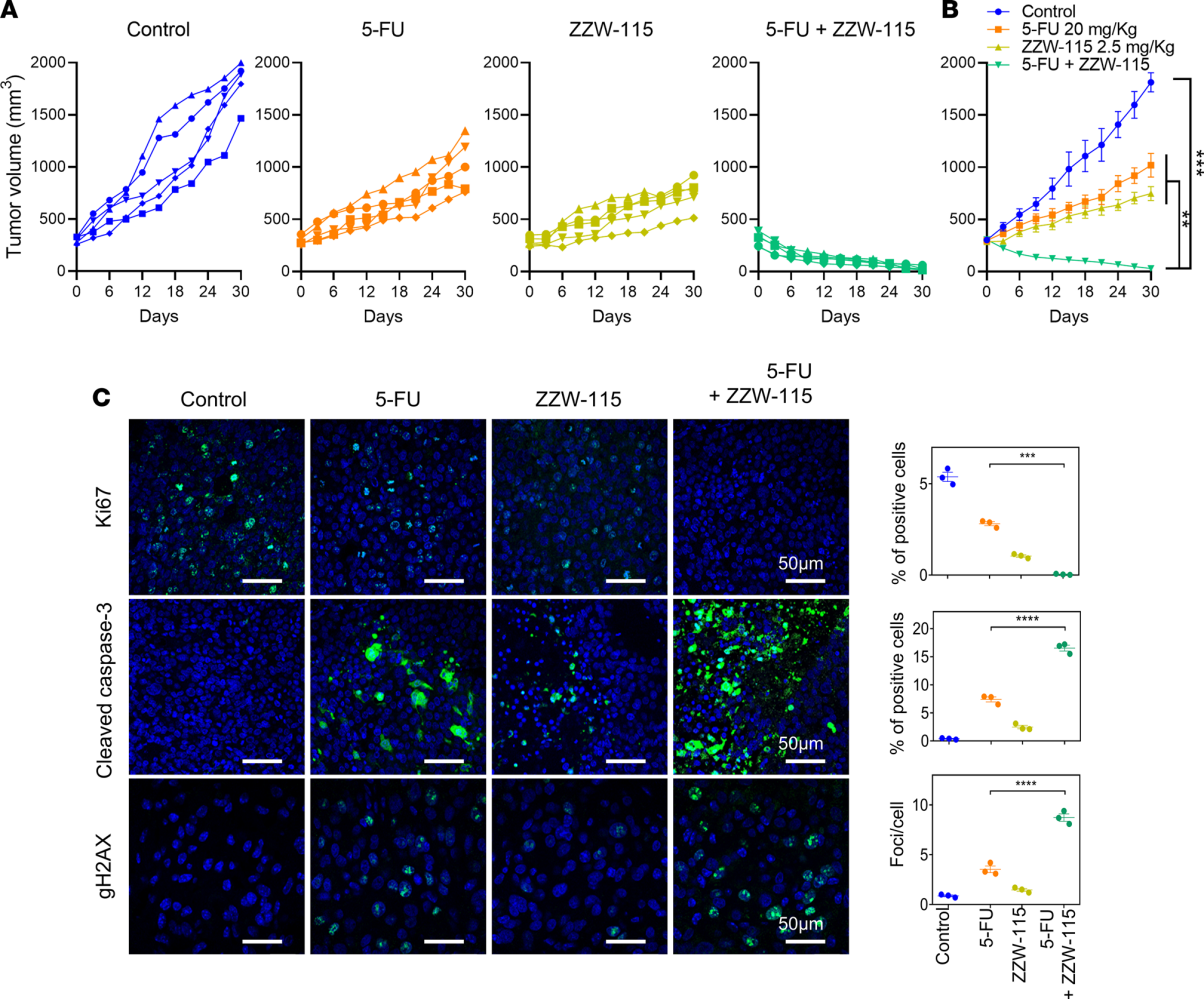
ZZW-115 strongly potentiated the antitumoral activity of on PDAC genotoxic agents in vivo. CAnN.Cg-Foxn1nu/Crl BALB/c nude mice xenografted with MiaPaCa-2 cells were separated into 4 groups of 5 mice and treated daily for 30 days with 0.5% DMSO in physiologic serum (control group), 20 mg/kg 5-FU, 2.5 mg/kg ZZW-115, or 20 mg/kg 5-FU in combination with 2.5 mg/kg ZZW-115. Tumor volume was measured every 3 days. (**A** and **B**) Individual volume of each mouse (**A**) and mean of the volume of each treatment (**B**) are shown. For each treatment, statistical significance is **P < 0.01 and ***P < 0.001 (1-way ANOVA, Tukey’s post hoc test). (**C**) Immunostaining of tumor samples with antibodies against Ki67, cleaved caspase-3, and γH2AX. Quantification of foci was performed by ImageJ software on 3 samples of each group. For each treatment, statistical significance is ***P < 0.001, ****P < 0.0001 (1-way ANOVA, Tukey’s post hoc test). Data represent mean ± SEM, *n* = 3.

MiaPaCa-2 xenograft grew in an exponential manner when treated with vehicle solution (from 326.64 ± 15.00 mm^3^ to 1813.60 ± 92.93 mm^3^ during 30 days). When mice were treated with 5-FU or ZZW-115 alone, the tumors grow from 298.81 ± 34.98 mm^3^ to 1019.19 ± 113.01 mm^3^ and from 345.43 ± 67.77 mm^3^ to 746.71 ± 67.64 mm^3^, respectively. Remarkably, the size of the tumors of all mice treated with the combination of 5-FU and ZZW-115 decreased immediately after the injections of the drug combination, until their disappearance (from 368.24 ± 45.00 mm^3^ to 28.48 ± 8.59 mm^3^) (Figure 4, A and B). To reveal the molecular mechanisms by which ZZW-115 exerts its effect on MiaPaCa-2, we performed immunofluorescence staining for γH2AX, cleaved caspase-3, and Ki67 to measure DNA damage, apoptosis, or antiproliferative effects. As shown in Figure 4C, tumors treated with the 5-FU/ZZW-115 combination displayed a large proportion of cells with DNA damage, greater number of apoptotic cells, and less proliferating cells — compared with single or no treatments.

### Glioblastoma.

When U87 tumors reached a volume of about 300 mm^3^, we started a daily treatment with 5 mg/kg of TMZ or ZZW-115 (2.5 mg/kg) either alone or in combination. The control group received the same volume of vehicle solution. Tumor volumes increased in an exponential manner in control mice (from 315.60 ± 6.25 mm^3^ to 1471.00 ± 176.80 mm^3^ during 21 days). When mice were treated with TMZ, the tumors had a slower development (from 334.30 ± 9.80 mm^3^ to 950.50 ± 290.85 mm^3^) (Supplemental Figure 3, A–C); tumors from mice treated with ZZW-115 also grew more slowly than in control mice (from 336.00 ± 28.10 mm^3^ to 903.50 ± 280.90 mm^3^). A significant intragroup variation was observed in both TMZ and ZZW-115 single-treated mice (Supplemental Figure 3, A–C). However, the size of the tumors of all mice treated with the combination of TMZ and ZZW-115 decreased immediately after starting the injections of the compounds and almost disappeared after 21 days of treatment (from 329.00 ± 27.00 mm^3^ to 24.30 ± 5.10 mm^3^). Results obtained for this group were much more homogenous since all tumors displayed a continuous regression until their complete disappearance (Supplemental Figure 3, A–C). Interestingly, we observed no tumor relapse in this group with combinatorial treatment, even after 25 days with no further treatment (data not shown). In addition, γH2AX, cleaved caspase-3, and Ki67 were stained to measure DNA damage, apoptosis, and antiproliferative effects, respectively. The tumors treated with the TMZ/ZZW-115 combination had a greater proportion of cells with DNA damage, large number of apoptotic cells, and less proliferating cells — compared with single treatments or control condition (Supplemental Figure 3D).

### NUPR1 localizes at DNA damaged sites and interacts with DNA repair proteins after treatment with DNA damaging agents.

**Figure 5:**
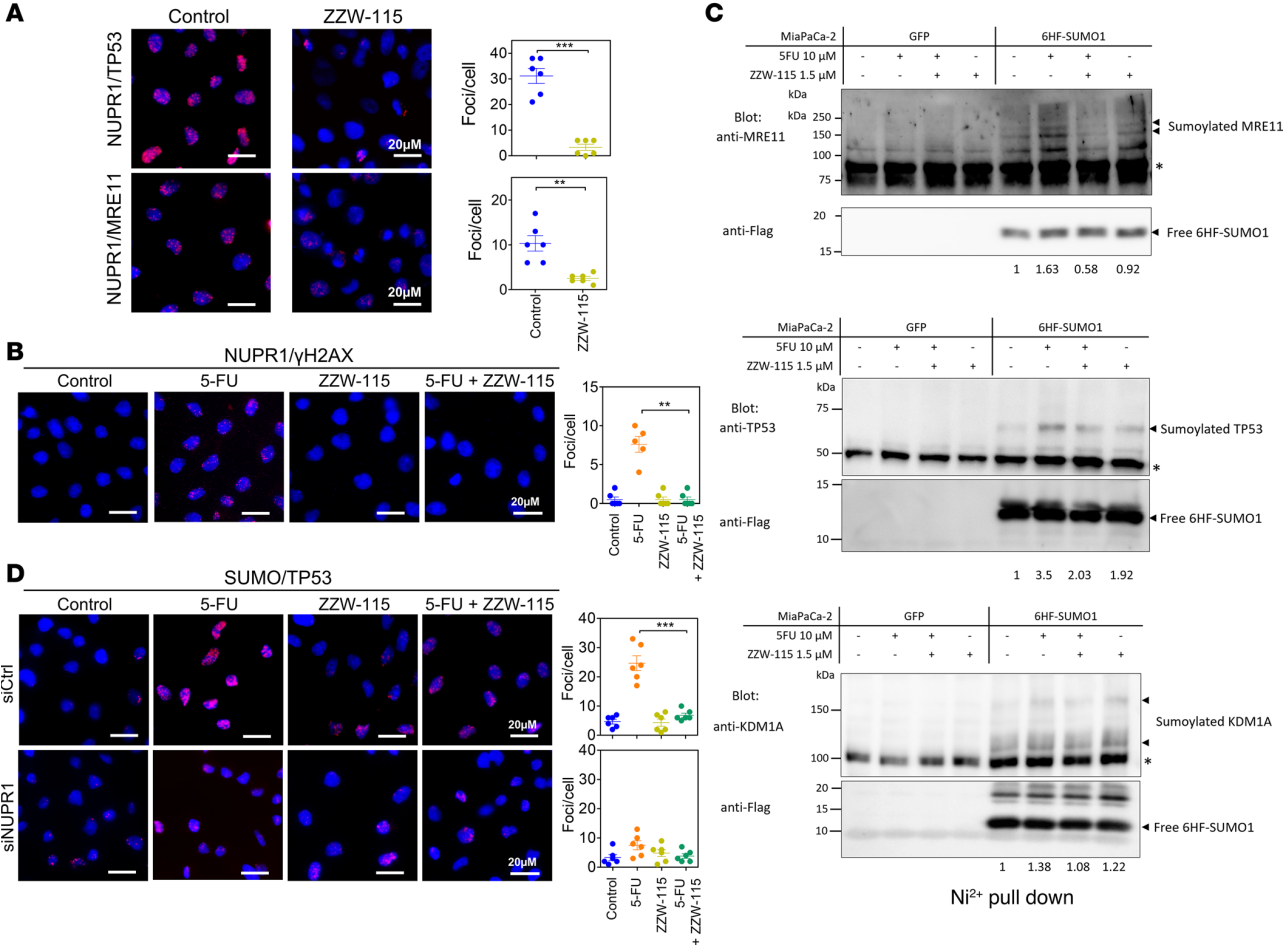
NUPR1 interacts with DNA repair proteins at DNA lesions, and its inhibition induces alterations of protein posttranslational modifications (PTM) profiles. (**A**) PLA was performed in MiaPaCa-2 cells, treated or not with ZZW-115 (5 μM) for 6 hours, transfected with a plasmid expressing NUPR1-Flag, using rabbit antibodies against MRE11 or TP53 and mouse anti-Flag. A representative experiment is shown (*n* = 3). ImageJ was used to count the number of red dots. Data represent mean ± SEM of 6 fields. Student’s 2-tailed unpaired *t* test was used. **P < 0.01; ***P < 0.001. (**B**) PLA was performed in MiaPaCa-2 cells (treated or no with ZZW-115 at 1.5 μM, 5-FU at 10 μM, or the combination of both for 24 hours) using rabbit anti-NUPR1 and mouse anti-γH2AX antibodies. Nontreated or treated with ZZW-11A representative experiment is shown (*n* = 3). ImageJ was used to count the number of red dots. Data represent mean ± SEM of 6 fields. One-way ANOVA with Tukey’s post hoc test was used. **P < 0.01. (**C**) Variations of SUMOylation of 3 identified DNA repair proteins and variations following the different treatments were studied by Western blot after purification of SUMOylated proteins in denaturing condition by Ni^2+^ pull-down. The upper parts of nitrocellulose filters were immunoblotted using anti-MRE11–, -TP53–, and -KDM1A–specific antibodies. Lower parts of filters were blotted using anti-Flag antibody in order to control the equal amount of precipitated material. Signals were quantified by densitometry, and values of the ratio of SUMOylated bands over precipitated SUMO1 are shown. Asterisk represents nonspecific bands (*n* = 1). (**D**) PLA was performed on MiaPaCa-2–6HF–SUMO1 cells transfected with siRNA against NUPR1 and treated with 5-FU and/or ZZW-115 (same conditions as mentioned above). SUMOylation of TP53 was quantified by mouse anti-Flag and rabbit anti-TP53 antibodies. A representative experiment is shown (*n* = 3). ImageJ was used to count the number of red dots. Data represent mean ± SEM of 6 fields. One-way ANOVA with Tukey’s post hoc test was used. ***P < 0.001.

Since pharmacological inhibition of NUPR1 exacerbates DNA damage, we were interested to probe if NUPR1 interacts with the DNA-repair proteins, which are recruited in these areas and were identified in the NUPR1’s interactome. In order to demonstrate that NUPR1 interacts with DNA repair proteins, we performed PLAs between NUPR1 and MRE11 or TP53. In both cases, positives PLA signals were observed in control conditions. However, as expected, incubation with ZZW-115 was capable of hampering this interaction (Figure 5A). Moreover, since NUPR1 interacts with DNA repair proteins, we demonstrated that NUPR1 is localized at the DNA lesions by performing a PLA between NUPR1 and the γH2AX (Figure 5B). Importantly, 5-FU treatment induced a greater interaction between both proteins, while this effect was almost completely reversed in the presence of ZZW-115. Taken together, these experiments demonstrate that NUPR1 interacts with DNA repair proteins and that it is localized at the DNA lesions.

### Analysis of protein PTMs intervening after treatments with 5-FU alone or in combination with ZZW-115.

Our previous results support the idea that NUPR1 was involved in DNA-repair processes induced by genotoxic agents. These DDR pathways rely on several posttranslational modifications (PTMs) of essential proteins to be activated and fully functional. Besides phosphorylation, PTMs mediated by ubiquitin and Ub-like members, such as SUMOs and Nedd8, were also shown to play crucial role in this process (19, 20). Therefore, we were interested in identifying changes within PTM profiles of main ubiquitin family members induced by a DNA damaging agent, 5-FU, alone or in combination with ZZW-115 in MiaPaCa-2 cells. Ubiquitinated, SUMOylated and Neddylated proteins were isolated from treated (see Methods) and untreated cells, and modified proteins were identified and semiquantified by MS.

A total of 1762 SUMOylated proteins were identified: 1404 in untreated cells, 450 alterations induced by 5-FU treatment (319 upward variations [up] and 131 downward variations [down]), 345 alterations induced by ZZW-115 treatment (136 up and 209 down), and 385 alterations induced by the cotreatment with both compounds (148 up and 237 down) (Table 3 and Supplemental Table 2). Interestingly, most 5-FU–induced SUMOylations were found to be inhibited when cotreated with ZZW-155 (Supplemental Table 3). Indeed, among the 319 proteins SUMOylated in response to the 5-FU treatment, only 2 seemed to be insensitive to the inhibitory effect of ZZW-155. A total of 1922 ubiquitinated proteins were identified, from which 1492 were found in untreated cells (Table 3 and Supplemental Table 4). Contrary to SUMOylations, 5-FU treatment induced a global decrease of ubiquitination rather than an increase, 345 and 133 proteins, with decreased and increased ubiquitinations, respectively). ZZW-115 induced mainly decreased ubiquitination (732 proteins) while only 58 proteins exhibited increased ubiquitination. Finally, the cotreatment with both drugs resulted in 460 and 150 proteins, with increased and decreased ubiquitinations, respectively (Table 3). The establishment of Nedd8-specific proteome led to the identification of 1696 Neddylated proteins, 1203 of which were found in untreated cells. Treatments with 5-FU, ZZW-115, or both molecules resulted mostly in increased Neddylations (285, 291, and 476 proteins, respectively) and a minority of deneddylations (114, 99 and 84 proteins, respectively) (Table 3 and Supplemental Table 5).

These inductions/repressions of PTMs induced by the different treatments were so important for some proteins that they could be observed only in the treated or nontreated groups (Supplemental Figure 4). We used the Panther GeneOntology online tool (http://www.pantherdb.org/) to study the biological processes mostly affected by the treatment with 5-FU (Supplemental Table 6). 5-FU induced alterations of SUMOylation; ubiquitination and Neddylations were involved in hundreds of different biological processes. However, when searching specifically for DNA-associated biological functions, we noticed that altered SUMOylations were involved in 6 of them, whereas altered ubiquitinations and Neddylations were involved in only 3. Most importantly, only alterations of SUMOylations displayed a specific enrichment in the DNA repair process. This enrichment contained 23 proteins with altered SUMOylations provoked by 5-FU, 14 of which with increased and 9 with decreased SUMOylations (Table 4). Noticeably, all of these 5-FU–induced SUMOylations were inhibited by ZZW-115 cotreatment (Table 4 and Supplemental Figure 5). Collectively, the proteomic data show that 5-FU treatment induced the hyper-SUMOylation of a number of proteins involved in the DDR, a process that was suppressed when the 5-FU treatment was combined with ZZW-115. We next aimed at validating these alterations of SUMOylations by specifically studying the behavior of some proteins among the identified targets. Using standard purification of SUMOylated proteins followed by Western blots, we could confirm as a proof of concept that the central DNA repair protein MRE11, TP53, and the histone demethylase KDM1A were all hyper-SUMOylated in response to 5-FU treatment and, importantly, that this response was efficiently inhibited by the cotreatment with ZZW-155 (Figure 5C).

Then, we studied whether inactivation of NUPR1 by a siRNA interference approach shows similar consequences as ZZW-115 treatment on the TP53 SUMOylation. To do this, we treated the MiaPaCa-2, stably transfected with SUMO1 (21), with siRNA against NUPR1 and then treated them with 5-FU. SUMOylation of TP53 was quantified by PLA using mouse anti-Flag and rabbit anti-TP53. As expected, SUMOylation was strongly improved after the treatment with 5-FU, but this increase was almost completely inhibited by ZZW-115. Treatment with siNUPR1 showed a dramatic inhibition of the SUMOylation as presented in Figure 5D. Altogether, our findings demonstrate that NUPR1 specifically takes part in the DNA repair process as it is involved in the mechanisms responsible for the increased SUMOylations, such as TP53, induced by a genotoxic agent like 5-FU.

### NUPR1 improved SUMOylation in a cell-free system.

**Figure 6:**
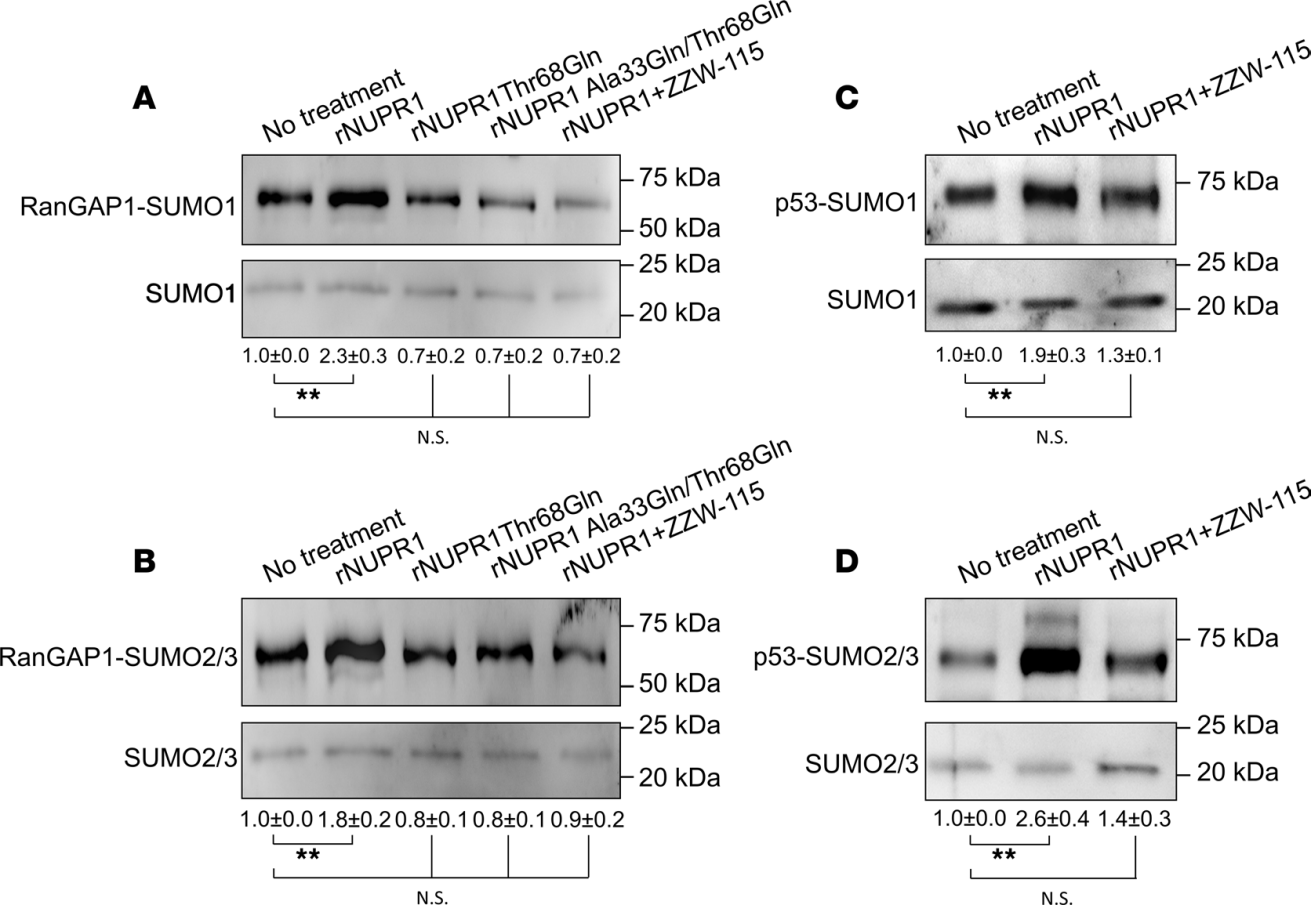
NUPR1 improved SUMOylation levels in vitro. (**A** and **B**) Western blot of in vitro SUMOylation by SUMO1 (**A**) or SUMO2/3 (**B**) of RanGAP1 was performed with no treatment or in the presence of recombinant WT rNUPR1, Thr68Gln, and Ala33Gln/Thr68Gln rNUPR1 mutants, as well as WT rNUPR1 in presence of ZZW-115. (**C** and **D**) Western blot analysis of in vitro SUMOylation by SUMO1 (**C**) or SUMO2/3 (**D**) of p53 was performed with no treatment or in the presence of recombinant NUPR1. **P < 0.01 compared with no treatment (1-way ANOVA, Tukey’s post hoc test). Data represent mean ± SEM, *n* = 4.

The interactome of NUPR1 revealed that it interacts directly or indirectly with UBC9 (the main SUMO conjugating enzyme), SUMO1, SUMO2/3, and RANBP2 (a major SUMO E3 ligase). Therefore, we hypothesized that NUPR1 could improve the SUMOylation of many proteins by acting as a stabilizer of the SUMOylation complex, mainly UBC9-SUMO and RANBP2. To test this possibility, we performed in vitro SUMOylation assays for SUMO1 and SUMO2/3, using recombinant RanGAP1 and p53 as substrates in the presence of: (a) recombinant WT NUPR1 (rNUPR1); (b) the recombinant Thr68Gln or Ala33Gln/Thr68Gln NUPR1 mutants; or (c) rNUPR1 in the presence of ZZW-115. Those 2 rNUPR1 mutants, with mutated residues belonging to either of the 2 hot-spot regions of NUPR1, have been designed to hamper interactions of NUPR1 with its natural partners (proteins or DNA) (17). The presence of WT rNUPR1 significantly increased the SUMOylation of the RanGAP1 and p53 substrates with both SUMO1 (Figure 6, A and C) and SUMO2/3 (Figure 6, B and D). Conversely, neither the presence of the mutants or, alternatively, NUPR1 together with ZZW-115 did increase the SUMOylation. Therefore, by interacting both with the SUMOylation machinery and with SUMOylation substrates, NUPR1 had the ability to stabilize the whole complex, thereby improving the SUMOylation of many proteins — some of which belong to the DDR.

## Discussion

Here, we describe the mechanism by which the previously designed bioactive compound ZZW-115 inhibits the nuclear activity of NUPR1. In particular we found that ZZW-115 interacts with NUPR1 through residue Thr68, which belongs to its predicted NLS sequence. The binding of the compound hampers the accessibility of the NLS to importin and then hinders the binding between the 2 biomolecules. We also found in vitro and in vivo that treatment of cells from several cancer types with ZZW-115 improved the anticancer activity of some, if not all, genotoxic agents, including radiations, by interfering with the SUMOylation of key proteins involved in the DDR.

### ZZW-115 shifted importins of the importin-cargo complex.

NUPR1 is a small IDP protein with a typical NLS sequence at its C-terminal region. Due to its disordered nature (and thus a large hydrodynamic radius), it needs to be transported from the cytoplasm to the nucleus to play its critical role for cancer cells. By using a proteomic-based strategy, we found that NUPR1 binds to proteins belonging to the nuclear pore complex (NPC). Our current knowledge of nuclear translocation indicates that the initial step of the classical nuclear transport process is the formation of the importin-cargo complex, in which importins bind cargo molecules after recognition of their NLS. Then, the N-terminus of importin-α (importin-β binding domain) binds to importin-β (22, 23). After the formation of the importin-cargo complex, importin-β is specifically recruited to NPC in the nuclear pore, and then the complex can pass through the nuclear pore. Each importin isoform has its own specific cargo molecules. We explored in vitro the binding between the importin-α3 isoform and NUPR1. The binding affected some of the aromatic residues of importin (as indicated by the changes in fluorescence), but it did not cause any change in the secondary structure of both proteins (as pinpointed by the absence of changes in CD spectra); NUPR1 remained disordered upon binding, forming a fuzzy complex (as it happens in the presence of other partner biomolecules) (15, 17, 18). The affinity of importin-α3 for NUPR1 was slightly greater (dissociation constant of 1.4 μM) than that of NUPR1 for ZZW-115 (4.7 × 10^5^ M) (12), although the enthalpy change associated with the former binding reaction was much larger (–13.7 kcal/mol versus –0.4 kcal/mol), likely reflecting more interactions established by NUPR1 with importin-α3, a 58 kDa protein, than with ZZW-115, a small molecule. As expected, the affinity of importin-α3 for NUPR1 was dramatically decreased in the presence of ZZW-115 (dissociation constant of 35 μM at 100 μM ZZW-115, Table 2). We then demonstrated, by using an independent and complementary approach named PLA, that NUPR1 interacted with importin-α3 in cellulo, in agreement with proteomic and biophysical analyses. Although importin-α3 and ZZW-115 have a similar affinity for NUPR1, ZZW-115 can shift the binding equilibrium between the 2 biomolecules (a) because of its smaller size (ZZW-115 binding to NUPR1 would be kinetically favored against that of NUPR1 to importin-α3); (b) if present at a comparable or higher concentration than that of importin-α3; and (c) if the potential ternary complex NUPR1/ZZW-115/importin-α3 is deficient regarding nuclear translocation. Altogether, these data show that ZZW-115 binds to the NLS region of the NUPR1 by partially shifting the binding equilibrium of NUPR1 with the importin and therefore acting as an inhibitor of its nuclear translocation.

Until recently, the palette of nuclear transport inhibitors had been limited to the inhibition of XPO1 by Leptomycin B (LMB) — which, however, failed during phase I clinical trials due to its toxicity (24). Since the discovery of LMB, an increasing number of new inhibitors of nuclear transport mediated by importins have been reported, but all retain their persistent toxicity due to their lack of specificity. On the contrary, a limited number of inhibitors that target specific cargos have been reported (25, 26). The first cargo-specific nuclear transport inhibitor described was mifepristone, a specific inhibitor of recognition of HIV-1 integrase by importin-α/β (27). There has been limited progress in the last few years in identifying and characterizing nuclear transport inhibitors due to their high toxicity. Since ZZW-115 specifically binds NUPR1 at residues involved in its NLS region, it shifts importins in a cargo-specific manner. We also assume that its toxicity, if any, will be low because there is no binding to the importin. In fact, thermal denaturations followed by CD indicate that the binding of ZZW-115 to importin-α3 does not take place (data not shown). Screening and identification of high-affinity compounds, using the NLS-specific cargos used as bait, could be a worthy strategy to identify new targets of nuclear proteins, as it has been suggested (25, 26, 28).

### NUPR1 promotes SUMOylation in response to genotoxic stress.

In response to DNA damage, induced by both genotoxic agents and radiation, cells activate a highly conserved and complex kinase-based signaling network, commonly referred to as the DDR, to safeguard genomic integrity. The DDR consists of a set of tightly regulated events, including detection of DNA damage and accumulation of DNA repair factors at the site of damage. The DDR imposes a cell cycle arrest to allow the DNA repair to take place and to preserve genome stability (29). Several PTMs occur during DDR (30), and among them, ubiquitinylation and SUMOylation have focused wide attention. Both Ubiquitylation and SUMOylation coordinate various pathways involved in DNA damage recognition and signaling and promote DNA repair (31). DNA repair factors have been identified to be SUMOylated and found to be enriched in the nuclear compartment after DNA damage (32, 33). SUMOylation regulates nuclear structures and is critical for multiple functions, including chromosome movement, centromeric functions, and DDR (34). Importantly, SUMO is thought to act synergistically on multiple proteins to create an environment favoring efficient DNA repair, probably by physically stabilizing the interactions between the proteins of the DNA repair machinery complexes (35). Recently, interplay between SUMOylation and phosphorylation in response to replication stress were shown to protect DNA integrity (36).

In this work, we show that 450 proteins were differentially SUMOylated in 5-FU–treated cells, and SUMOylation of many of them was reversed by the addition of ZZW-115, indicating that NUPR1 plays a major role in controlling SUMOylation after 5-FU treatment. Importantly, a substantial amount of these proteins is involved in the DDR, and many of them are known to be regulated by SUMOylation (Table 4). This general effect of NUPR1 was confirmed on 3 targets involved in the DDR (MRE11, TP53, and KDM1A), for which hyper-SUMOylated in response to 5-FU treatment was inhibited by the cotreatment with ZZW-155 (Figure 5C). Although MRE11, TP53, and KDM1A play important roles in the DDR, the role of their SUMOylation in response to DNA damage has not yet been reported. TP53 has been shown to be SUMOylated (37, 38), although the consequences of its SUMOylation remains unclear (39). KMT1 and yeast Mre11 have been both shown noncovalently linked with SUMO1 (40, 41). Our results suggest that they are linked covalently to SUMO after genotoxic treatment. Notably, we confirmed that SUMOylation of proteins involved in DDR depends on NUPR1 activity, since its knocking down with specific siRNA decreased the SUMOylation of the TP53 after DDR induced with 5-FU (Figure 5D).

SUMOylation assay performed in a cell-free system showed that rNUPR1, but not Thr68Gln and Ala33Gln/Thr68Gln mutants (involving residues belonging to the hot-spot regions of NUPR1), enhanced the SUMOylation of recombinant RanGAP1 and p53 (Figure 6). The absence of enhancement in the SUMOylation by the mutants is probably due to the fact that complex formation involves the same 2 regions of NUPR1 (residues around Ala33 and Thr68), which also intervene in binding with other proteins. Importantly, adding ZZW-115 during the complex formation inhibited the effect of rNUPR1, by binding to Thr68 and Ala33 (causing the same effect as the 2 mutations). Altogether, we can assume, although it was not formally demonstrated, that NUPR1 acted as a general facilitator of the SUMOylation by binding and stabilizing the complex formed between the SUMOylation machinery and the substrate proteins (i.e., to the complex formed by several proteins), as suggested by the NUPR1 interactome (Table 1). Interestingly, 5-FU cotreatment with ZZW-115 induced mainly an increase in ubiquitinylations and many decreased SUMOylations (Table 3), suggesting a stimulation of the ubiquitin-dependent protein degradation.

### ZZW-115 sensitizes cancer cells to DNA damage–based radio and chemo therapies.

The success of the use of physical and chemical DNA damaging agents as anticancer therapeutics is based, and mainly relies, on the uncontrolled proliferation of cancer cells. Indeed, many genotoxic agents target the replication stage of the cell cycle, when cells need to duplicate their genome, to control and guarantee the quality of the produced new DNA. Therefore, the efficacy of such treatments is further enhanced in cancer cells containing a defect in DDR. However, many tumors are, or become, resistant to these kinds of therapies. These resistance mechanisms include, among others, reduced uptake of the drug by the tumor cells, an increased intracellular or systemic inactivation of the drug, a general resistance to stress-induced cell death, and the increased efficiency of DNA repair mechanisms. Therefore, some strategies have the aim to lower the resistance of cancer cells to genotoxic agents. Most of these potentiating molecules, but not all, do not target directly the DNA damage repair mechanisms but target pathways involved in cell survival/death or aim at increasing the uptake, activation, and stability of the genotoxic drugs. Hence, these molecules are having a more additive effect than a potentiating outcome on the genotoxic agent. Here, we propose that specific inhibition of NUPR1 function by ZZW-115 displayed a strong potentiating effect of several genotoxic agents and on diverse cancer cell types by directly impairing the SUMOylation (Figure 5) of the proper function of the major DNA repair actors (Figure 3) or their molecular partners. We do not formally rule out that ZZW-115 could exert its anticancer activity by preventing the binding of NUPR1 to factors involved in RNA splicing and processing; however, the fact that ZZW-115 has a synergic effect mainly with genotoxic agents and that it inhibits 5-FU–induced SUMOylation rather support our model. We therefore presume that ZZW-115 decreases the SUMOylation by targeting NUPR1, which in turn decreases DNA repair and consequently increases cell death. In this work, we do not demonstrate that a direct effect of inhibition of NUPR1-dependent SUMOylation by ZZW-115 is beneficial to anticancer activity — rather, we demonstrate its effect on SUMOylation. However, in the literature, the work of Biederstadt and colleagues (42) shows that inhibition of SUMOylation by small compounds has a strong effect on aggressive PDAC, indicating that SUMOylation is an essential process for PDAC development and survival. Taken together, we suggest that ZZW-115 treatment, as well as inhibition of NUPR1 by siRNA, promotes cell death in response to DNA damage by inhibiting SUMOylation.

Other molecules addressing — more specifically — the DDR, such as PARP inhibitors, cell cycle checkpoint inhibitors, or proteins involved in base excision repair or in double-strand break (DSB) repair, have been shown to sensitize cancer cells to radiotherapy (43). However, because DNA repair mechanisms are also effective in noncancerous cells and are necessary to warranty their genome stability, they might have serious side effects for the patients. Hence, one advantage of a compound like ZZW-115 is that it potentiates the effect of genotoxic agents by targeting a stress-induced protein, NUPR1, which is abnormally present in many cancer cells but almost absent from normal cells. In this way, we confirmed that ZZW-115 was unable to modify the effect on DNA damage induced by the genotoxic agent 5-FU in human nontransformed fibroblasts (Supplemental Figure 2). Future use in other animal models, and hopefully clinical trials, should confirm the low systemic toxicity of NUPR1 inhibition as we observed in xenografted mice cotreated with TMZ or 5-FU and ZZW-115. Indeed, no general toxicity was observed in the mice, whereas their xenografted tumors completely disappeared (Figure 4 and Supplemental Figure 3).

### Conclusion.

To sum up, ZZW-115 interacts with the NLS sequence of NUPR1 with similar affinity as that of importins for the protein and is able to interfere with the binding reaction between importin-α3 and NUPR1, hampering the nuclear translocation of the latter. Treatment with ZZW-115 enhanced the DNA damage induced by genotoxic agents, both in vivo and in vitro*,* through significantly altering the PTM profiles, mainly SUMOylation, and therefore affecting the DDR. ZZW-115 is a promising candidate to be used in combination with genotoxic agents potentially in the treatment of most cancers.

## Methods

### Flag-NUPR1 coimmunoprecipitation.

MiaPaCa-2 cells, expressing Flag-NUPR1 or Flag-GFP, were plated in 10 cm^2^ dishes. When MiaPaCa-2 cells expressing Flag-NUPR1 or Flag-GFP reached 70% confluence, they were lysed on ice by using HEPES-based lysis buffer containing 10 mM NEM (N-Ethylmaleimide, MilliporeSigma, 04259) and a proteases inhibitor cocktail (1:200) (MilliporeSigma, P8340). Lysates were centrifuged for 10 minutes at 21,000*g* at 4°C. Protein concentration of the supernatant was determined by using Protein Assay (Bio-Rad), and equal amounts of total protein were used to incubate with 30 μL of anti–Flag M2–coated beads (MilliporeSigma, F3165) under rotation for 2 hours at 4°C. Beads were then washed 3 times with cold lysis buffer, and proteins were eluted using 250 μL ammonium hydrogen carbonate buffer containing 0.1 μg/μL of Flag peptide (MilliporeSigma, F3290) for 90 minutes at 4°C while rotating. After a short spin, the supernatant was recovered by using a Hamilton syringe. Eluted proteins were collected and analyzed by MS.

### Lentiviral infection of MiaPaCa-2 cells with 6His-Flag-ubiquitin–like constructs.

A tandem 6His and Flag tag was introduced into empty pCCL-WPS-mPGK lentiviral vector, at the 5′ end of the multicloning sites portion, to produce the pCCL-6HF vector. The full-length cDNA for human ubiquitin, Nedd8, and SUMO1 were subcloned into this vector using SmaI and EcoRV restriction sites for ubiquitin, BamHI, and EcoRV for Nedd8, and BamHI for SUMO1. Lentiviral particles were generated by transfecting 293T cells with a mix of 1/3 pCCL construct (Ub, Nedd8, SUMO1, or GFP), 1/3 delta Helper and 1/3 pVsVg, using Lipofectamine reagent (Invitrogen) and following manufacturer’s recommendations. Beyond 24 hours after transfection, the medium was changed with a fresh one. After another 24 hours, medium was changed again and viruses contained in the medium were collected, filtered through a 0.2 μm filter, and added on 40% confluent MiaPaCa-2 cells seeded in 25 cm^2^ flasks. This step was repeated 24 hours later to perform a second infection.

### Two-step purification of 6His-Flag-ubiquitin, -Nedd8, and -SUMO1 conjugates.

MiaPaCa-2 cells expressing the 6His-Flag-ubiquitin–like constructs or GFP were seeded in 150 mm dishes, at 1 × 10^6^ cells per dish, and when they reached 70% confluence, they were treated with 5-FU or ZZW-115 or the combination of both drugs for 12 hours. Then, approximately 100 (MiaPaCa-2–6His–Flag–ubiquitin and –SUMO1) or 150 mg (MiaPaCa-2–6His–Flag–Nedd8) of proteins were used to isolate modified substrates. For each dish of MiaPaCa-2 cells with different treatment conditions, 2 mL of buffer 1 (6 M guanidinium chloride, 0.1 M Na_2_HPO_4_/ NaH_2_PO_4_, pH 8.0 plus 0.5% Triton X-100; Sigma-Aldrich, T8787) were added directly to the cell monolayer. Lysates were sonicated 3 times for 30 seconds with a 1-minute break between pulses, to reduce viscosity. Protein concentration was measured in untreated and treated samples, and Ni^2+^-NTA agarose resin (QIAGEN, 30210) was added with a ratio of 2 μL of resin for 1 mg of proteins. Samples were rotated at room temperature for 2 hours an 30 minutes, and beads were then washed once with 1 mL of buffer 1 and twice with 1 mL of prechilled buffer 2 (50 mM NaH_2_PO_4_, 150 mM NaCl, 1% Tween-20, 5% glycerol, pH 8.0) plus 10 mM imidazole. Purified proteins were eluted after 2 hours at 4°C in 600 μL of buffer 2 plus 250 mM imidazole. Eluted proteins were then incubated with 50 μL of anti–Flag M2 agarose beads (MilliporeSigma, F3165) and rotated at 4°C for 2 hours and 30 minutes. Beads were then washed twice with 500 μL of prechilled buffer 2. Purified proteins were eluted in 100 μL of buffer 2 containing 0.1 μg/μL of Flag peptide (MilliporeSigma, F3290) by rotating at 4°C for 1 hour and 30 minutes. Eluted proteins were collected and analyzed by MS.

### One step Ni^2+^ purification.

Like the 2-step procedure but without the Flag purification and with little modifications, 5 mL of guanidinium lysis buffer and 50 μL of Ni^2+^-NTA beads were used per 15 cm diameter dishes of cell culture. After washing with 8 M Urea buffers (pH8 then pH6.3), the bound proteins were eluted using 1× Laemmli buffer containing 200 mM imidazole for 5 minutes at 95°C. For cell lysate, a fraction of the guanidinium lysate was precipitated using ethanol procedure and proteins dissolved in 1× Laemmli buffer for 5 minutes at 95°C.

### MS analysis.

Protein extracts were loaded on NuPAGE 4%−12% Bis-Tris acrylamide gels according to the manufacturer’s instructions (Invitrogen). Running was stopped as soon as proteins stacked in a single band. Protein-containing bands were stained with Imperial Blue (Pierce), cut from the gel, and digested with high-sequencing-grade trypsin (Promega) before MS analysis according to Shevchenko et al. (44). MS analysis was carried out by LC-MS/MS using an LTQ-Velos-Orbitrap or a Q Exactive Plus Hybrid Quadrupole-Orbitrap (Thermo Fisher Scientific) coupled online with a nanoLC Ultimate3000RSLC chromatography system (Dionex). A total of 5 μL corresponding to one-fifth of the whole sample were injected in triplicate on the system. After sample preconcentration and washing on a Dionex Acclaim PepMap 100 C18 column (2 cm × 100 μm i.d. 100 Å, 5 μm particle size), peptides were separated on a Dionex Acclaim PepMap RSLC C18 column (15 cm × 75 μm i.d., 100 Å, 2 μm particle size) at a flow rate of 300 nL/min, a 2-step linear gradient (4%−20% acetonitrile/H2O; 0.1% formic acid for 90 minutes and 20%−45% acetonitrile/H_2_O; 0.1% formic acid for 30 minutes). For peptide ionization in the nanospray source, voltage was set at 1.9 kV, and the capillary temperature was set at 275°C. All samples were measured in a data-dependent acquisition mode. Each experiment was preceded by a blank run to monitor system background. The peptide masses were measured in the LTQ-velos-orbitrap in a survey full scan (scan range 300−1700 *m/z*, with 30 K FWHM resolution at *m/z* = 400, target AGC value of 1 × 10^6^, and maximum injection time of 200 ms). In parallel to the high-resolution full scan in the Orbitrap, the data-dependent collision-induced dissociation (CID) scans of the 10 most intense precursor ions were fragmented and measured in the linear ion trap (normalized collision energy of 35%, activation time of 10 ms, target AGC value of 1 × 10^4^, maximum injection time 100 ms, and isolation window 2 Da). Parent masses obtained in Orbitrap analyzer were automatically calibrated on 445.1200 locked mass. Dynamic exclusion was implemented with a repeat count of 1 and exclusion time of 30 seconds.

In the Q Exactive Plus Hybrid Quadrupole-Orbitrap, the peptide masses were measured in a survey full scan (scan range 375–1500 *m/z*, with 70 K FWHM resolution at *m/z* = 400, target AGC value of 3 × 10^6^ and maximum injection time of 100 ms). Following the high-resolution full scan in the Orbitrap, the 10 most intense data-dependent precursor ions were successively fragmented in higher-energy collisional dissociation (HCD) cell and measured in Orbitrap (normalized collision energy of 25%, activation time of 10 ms, target AGC value of 1 × 10^3^, intensity threshold 1 × 10^4^ maximum injection time 100 ms, isolation window 2 *m/z*, 17.5 K FWHM resolution, scan range 200–2000 *m/z*). Dynamic exclusion was implemented with a repeat count of 1 and exclusion time of 20 seconds.

### MS data analysis.

Raw files generated from MS analysis were processed using Proteome Discoverer 1.4.1.14 (Thermo Fisher Scientific). This software was used to search data via in-house Mascot server (version 2.3.0; Matrix Science) against the Human database subset of the SwissProt database (version 2017.03, 20184 human entries). A database search was done by using the following settings: a maximum of 2 trypsin miscleavage allowed, methionine oxidation and protein N-acetylation as dynamic modifications, and cysteine carbamido-methylation as fixed modification. A peptide mass tolerance of 6 ppm and a fragment mass tolerance of 0.8 Da were allowed for search analysis. Only peptides identified with a FDR < 1% were used for protein identification.

### Protein expression.

NUPR1 was expressed and purified as described (12). A codon-optimized vector containing residues 1–520 of importin-α3 was synthetically produced by Nzytech. The protein was expressed overnight in BL21 *E*. *coli* strain at 37°C, in LB medium, after induction with 1 mM (final concentration) of IPTG (isopropylthio-galacto-pyranoside), when the culture had reached an absorbance at 600 nm between 0.6 and 1.0. Purification was similar to that described for NUPR1 (12), except that the final polish purification step was carried out with a Superdex G200 16/60 in buffer Tris (50 mM, pH 8.0) with 200 mM NaCl, running on an AKTA FPLC (GE Healthcare) by following the absorbance at 280 nm.

### CD and fluorescence spectroscopies.

The experimental set-up for CD and fluorescence spectroscopies was the same described previously (12). The temperature was 25°C for both techniques, and protein concentrations were 10 μM of NUPR1 and 5 μM of importin-α3 for the fluorescence experiments, and 20 μM of NUPR1 and 5 μM of importin-α3 for the CD experiments.

### ITC.

The experimental set-up and data processing of ITC experiments has been described previously (12). Importin-α3 (100–110 μM) was loaded into the syringe, and NUPR1 (5–10 μM) into the calorimetric cell in buffer Tris 50 mM, pH 8. Reverse titrations (NUPR1 in the syringe and importin-α3 in the calorimetric cells) were also carried out, but direct and reverse titrations provided similar thermodynamic binding parameters. The temperature for all the experiments was 25°C. The binary experiments (interaction of NUPR1 with importin-α3) were analyzed applying a model considering a single binding site (1:1 stoichiometry for the NUPR1/importin-α3 interaction). For experiments in the presence of ZZW-115, a concentration of the compound of 100 μM was kept constant during the titration. These ternary experiments were analyzed in 2 ways: (a) considering an apparent quasibinary system with a single binding site displaying apparent thermodynamic binding parameters implicitly dependent on the concentration of ZZW-115 and (b) considering an exact ternary system with a single binding site displaying intrinsic thermodynamic binding parameters explicitly dependent on the concentration of ZZW-115 through cooperative interaction parameters (45, 46). The concentration of ZZW-115 in the calorimetric cell was much higher than its dissociation constant for its interaction with NUPR1, and the data analysis with both models gave similar values for the reduction in affinity for the NUPR1/importin-α3 interaction caused by the presence of ZZW-115.

### PLA.

MiaPaCa-2 cells were seeded on coverslips and transfected with 2 μg of plasmid DNA (Nupr1-Flag or importin-α3–Flag) using Lipofectamine 3000 Transfection Reagent (Thermo Fisher Scientific). MiaPaCa-2–6HF–SUMO1 cells, previously described (21), were used to measure the SUMOylation of TP53. At the end of the experiment, cells were washed in PBS, fixed, and permeabilized before immunostaining with Duolink In Situ (MilliporeSigma) following the manufacturer’s protocol. Anti-NUPR1 (rabbit, homemade), -p53 (rabbit, Santa Cruz Biotechnology, sc-6243), -MRE11 (rabbit, Novus Biologicals, NB100-142), or -Flag (mouse, MilliporeSigma, F1804) were used. Image acquisition was carried out on a Nikon Eclipse 90i fluorescence microscope. ImageJ (NIH) was used to count the number of red dots.

### Use of genotoxic agents.

DNA breaks within different cancer cell lines (U87 and U251, glioblastoma cells; MiaPaCa-2, PDAC cell line; AOIPC, PDAC patient–derived cells; HT29, colon carcinoma; and the boosting effect of ZZW-115) was evaluated by γH2AX immunofluorescence staining. Cells were treated with TMZ (180 μM), 5-FU (10 μM), Gemcitabine (15 μM), Oxaliplatin (12.5 μM), or γ-radiation (6 Gy) alone or in combination with ZZW-115 (1.5 μM), and DNA damages were quantified after 12 hours. Human PDAC–associated fibroblasts were used as nontransformed cells. Sorafenib (0.5 μM) was used on HepG2 (hepatocarcinoma) cells as a negative control since it does not induce DNA damage.

### Western blotting.

Proteins were resolved by SDS-PAGE, and transferred to nitrocellulose membranes for 1 hour. Then, membranes were blocked 1 hour at room temperature with TBS (Tris buffered saline solution) and 5% BSA, and blotted overnight in TBS 5% BSA containing primary antibodies (1:500; anti-p53 [rabbit, Santa Cruz Biotechnology Inc., sc-6243], -MRE11 [rabbit, Novus Biologicals, NB100-142], -LSD1 [mouse, Santa Cruz Biotechnology Inc., sc-271720]). After extensive washes in TBS 0.1% Tween-20, membranes were incubated 1 hour at room temperature with HRP-conjugated secondary antibodies at 1:5000 before being revealed with Enhanced chemo-luminescence (ECL). Acquisition was performed with a Fusion FX7 imager (Vilber-Lourmat).

### Animals.

Female CAnN.Cg-Foxn1^nu^/Crl BALB/c nude mice were provided by Charles River Laboratories. Mice were kept within the Experimental Animal House of the Centre de Cancérologie de Marseille, pôle Luminy (CRCM). Ten million MiaPaCa-2 cells or U87-red glioblastoma were inoculated s.c. in nude mice (6 weeks old), and they were separated into 4 groups of 5 or into 4 groups of 6 subjects each, respectively. Mice injected with MiaPaCa-2 cells were treated with 0.5% DMSO in physiologic serum (vehicle), 20 mg/kg 5-FU (5 days per week), 2.5 mg/kg of ZZW-115 (daily), and a combination of 20 mg/kg 5-FU with 2.5 mg/kg ZZW-115 when the tumor volume reached 300 mm^3^. Mice injected with U87-red glioblastoma were treated daily with 0.5% DMSO in physiologic serum (vehicle), 5 mg/kg of TMZ, 2.5 mg/kg of ZZW-115, and a combination of 5 mg/kg TMZ with 2.5 mg/kg ZZW-115 when the tumor volume reached 300 mm^3^. Mice with MiaPaCa-2 xenografts were sacrificed after 30 days of treatment. Every 3 days, the mice were weighed and the tumor volumes were measured. Mice with U87 xenografts were sacrificed after 21 days of treatment, except 3 mice from the combination group. These 3 mice were kept for an additional 25-day period without any additional treatment.

### Immunofluorescence of cultured cells.

Cells were seeded in 12-well plates on coverslips and treated with ZZW-115. After fixation, cells were incubated with the following antibodies at 1:100 dilution: rabbit anti-NUPR1 primary antibody (homemade) or γH2AX primary antibody (ab26350, Abcam). After washing steps, samples were incubated in the presence of secondary antibodies at 1:200 dilution (goat anti–mouse Alexa Fluor 488, A28175, or goat anti–rabbit Alexa Fluor 488, A27034, both from Thermo Fisher Scientific). DAPI (D1306, Thermo Fisher Scientific) was used to stain the nucleus. Image acquisition of Alexa Fluor 488–derived fluorescence and DAPI staining was performed using an LSM 880 controlled by Zeiss Zen Black. Colocalization analysis and measurement of both channels was conducted by using the ImageJ Coloc 2 plugin.

### Immunofluorescence staining of tumor samples.

Immunofluorescence staining was performed on 5 μm–thick paraffin-embedded tissue sections. The following antibodies were used: Ki67 (ab92742), γH2AX (ab26350) (both from Abcam), cleaved caspase-3 (9661; Cell Signaling Technology), and anti-NUPR1 (homemade). Primary antibodies were diluted 1:200 and secondary antibodies (goat anti-mouse IgG [H+L] Alexa Fluor 488 [A28175] and goat anti-rabbit IgG [H+L] Alexa Fluor 488 [A27034], both from Thermo Fisher Scientific) were diluted 1:500. Signals were detected with an LSM 880 controlled by Zeiss Zen Black. Colocalization analysis and measurement of both channels was performed by ImageJ software.

### In vitro (cell-free) SUMOylation assay.

In vitro SUMO assay was performed using a SUMOylation kit by Enzo Life Sciences according to manufacturer’s protocol. Reactions containing SUMO1, SUMO2, and SUMO3; E1 or E2 (Ubc9); and RanGAP1 or p53 in the presence of recombinant WT NUPR1 (2 μM), Thr68Gln (2 μM), and Ala33Gln/Thr68Gln (2 μM) NUPR1 mutants or in the presence of NUPR1 with ZZW-115 (100 μM) were incubated 1 hour at 37°C. SUMOylation of RanGAP1 or p53 were analyzed by Western blotting using rabbit anti-SUMO1 or SUMO2/3 antibodies provided by the kit (BML-UW8955; SUMO-1, rabbit polyclonal antibody, BML-PW8330; SUMO-2/3, rabbit polyclonal antibody, BML-PW9465).

### Statistics.

Statistical analyses were performed by using the unpaired 2-tailed Student *t* test or 1-way ANOVA with Tukey’s post hoc test. Values are expressed as mean ± SEM. Data are representative of at least 3 independent experiments with technical triplicates completed. A *P* value less than 0.05 was considered significant.

### Study approval.

All experimental protocols were carried out in accordance with the *Guide for the Care and Use of Laboratory Animals* (National Academies Press, 2011). All experimental procedures on animals were approved by the Comité d’éthique de Marseille numéro 14 (C2EA-14).

## Author contributions

WL, PSC, MS, ZZ, SA, LC, CH, AJA, PS, AVC, OA, BR, and JLN conducted experiments. WL, PSC, MS, YX, LP, BR, JLN, GL, RU, and JI contributed to study design and reviewed and revised the manuscript. PSC, PS, VG, JLN, and JI analyzed and interpreted the data. PSC, YX, VG, PS, BR, JLN, and JI wrote the manuscript. JI designed and supervised the study.

## Supplementary Material

Supplemental data

Supplemental Table 1

Supplemental Table 2

Supplemental Table 3

Supplemental Table 4

Supplemental Table 5

Supplemental Table 6

## Figures and Tables

**Table 4:**
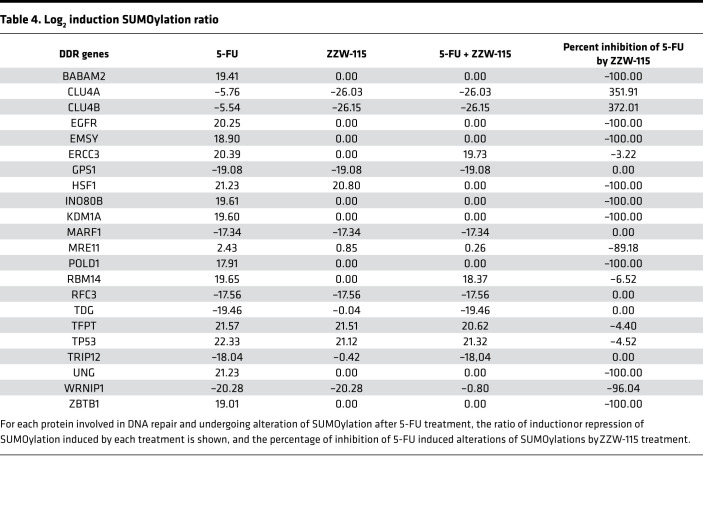
Log_2_ induction SUMOylation ratio

**Table 3:**
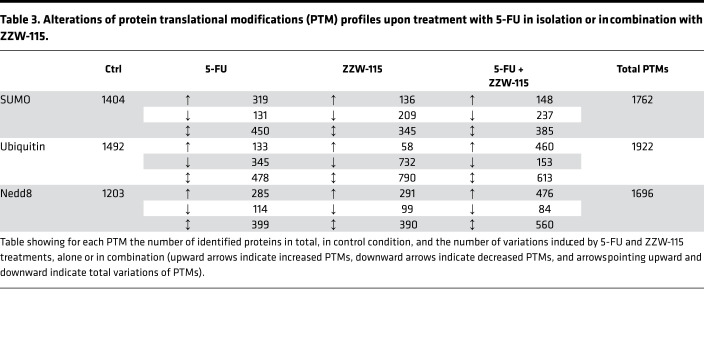
Alterations of protein translational modifications (PTM) profiles upon treatment with 5-FU in isolation or in combination with ZZW-115.

**Table 1:**
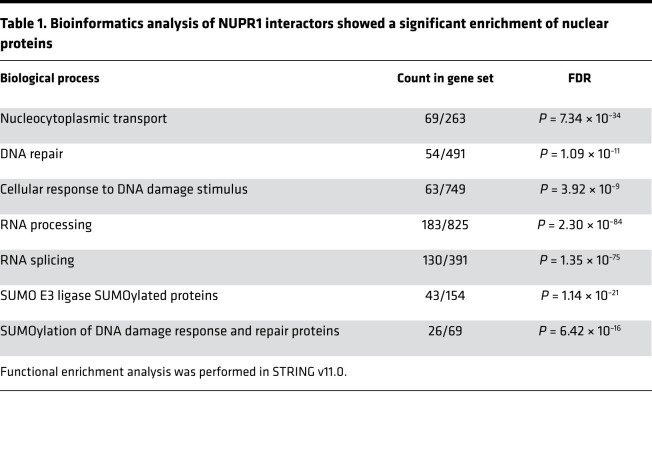
Bioinformatics analysis of NUPR1 interactors showed a significant enrichment of nuclear proteins

**Table 2:**
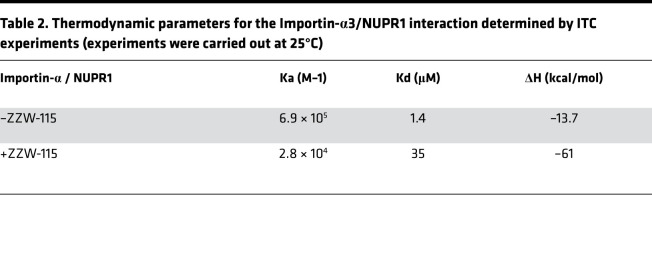
Thermodynamic parameters for the Importin-α3/NUPR1 interaction determined by ITC experiments (experiments were carried out at 25°C)

## Version 1 08/11/2020

In-Press Preview

## Version 2 09/17/2020

Electronic publication
