# Supplementary material for: ZZW-115–dependent inhibition of NUPR1 nuclear translocation sensitizes cancer cells to genotoxic agents
Source: JCI Insight. 2020 Sep 17;5(18):e138117. doi: 10.1172/jci.insight.138117 (PMC7526551; doi:10.1172/jci.insight.138117)
Supplement: Supplemental data [file jciinsight-5-138117-s191.pdf]

Legends of Supplementary Figures

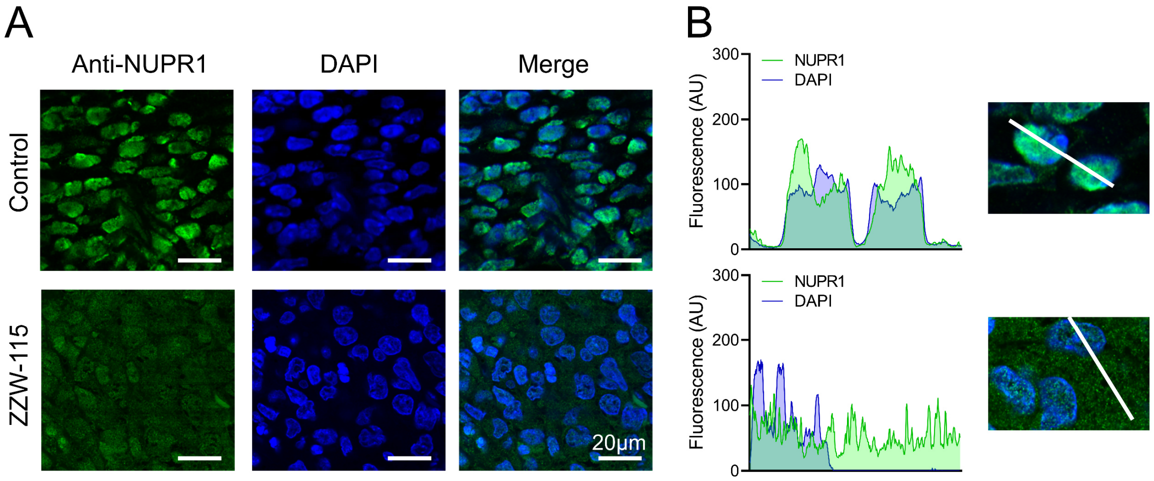

Supplementary Figure 1

ZZW-115 inhibited NUPR1 nuclear translocation *in vivo*. Xenografts of MiaPaCa-2 cells were treated with ZZW-115 5 mg/kg/day for 30 days. Immunofluorescence with rabbit anti-NUPR1 primary antibody and Alexa 488-labeled goat anti-rabbit secondary antibody were used to reveal the localization of the protein. DAPI staining was used to detect cell nuclei. (Magnification: 63x). (B) Intensity profiles along the white line in the image are shown. A representative experiment is shown (n = 3).

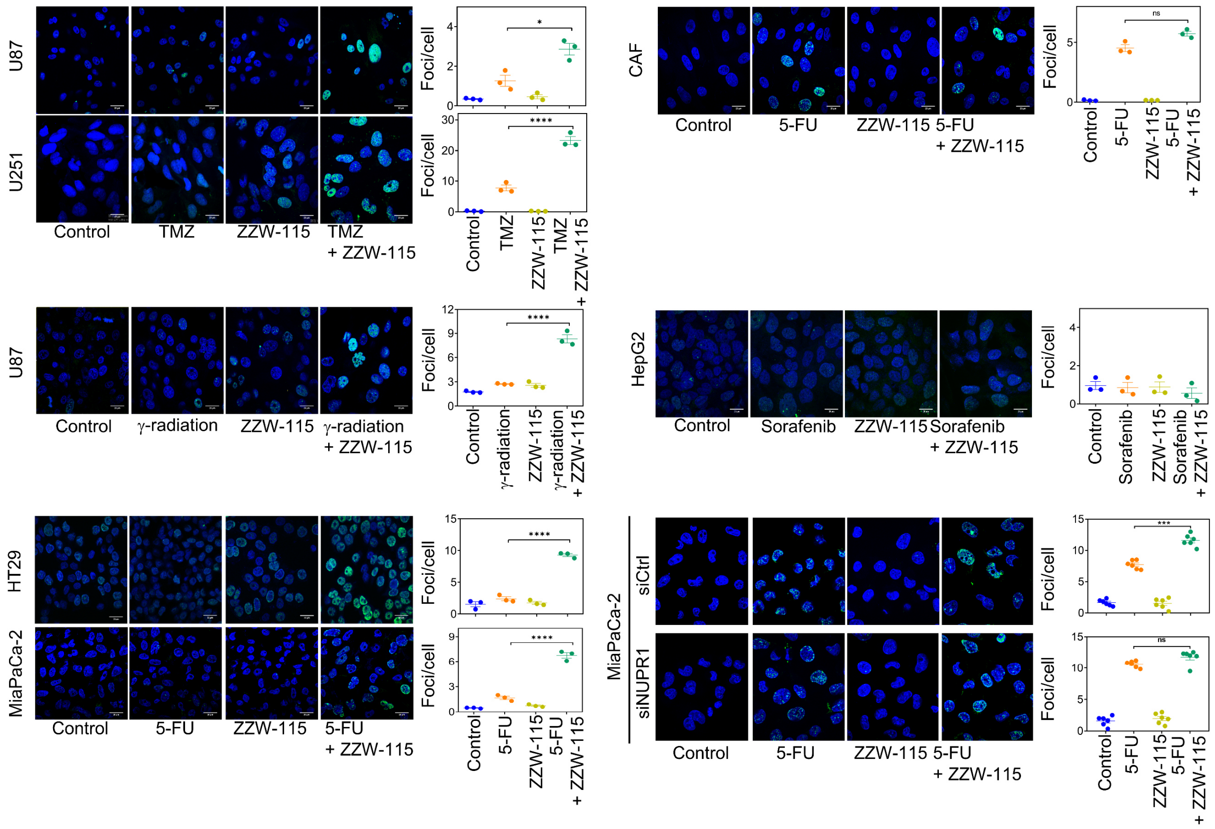

Supplementary Figure 2

NUPR1 inhibition by ZZW-115 potentiated the efficacy of genotoxic agents in several cell lines. The efficacy of different genotoxic agents (TMZ, 5-FU, and γ-radiation) to generate DNA breaks in U87 and U251 (glioblastoma), HT29 (colon carcinoma), cancer associated fibroblasts (CAF) and HepG2 (hepatocarcinoma) cell lines and the boosting effect of ZZW-115 was evaluated by γH2AX immunofluorescence staining. MiaPaCa-2 cells were transfected with siCtrl or siNUPR1 and treated with 5-FU and/or ZZW-115 and DNA damage was evaluated by γH2AX immunofluorescence staining. p-value < 0.05 *; 0.0001 **** (one-way ANOVA, Tukey's post hoc test). Data represent mean ± SEM, n = 3. Sorafenib was used as negative control since it does not induce DNA damage. TMZ: Temozolomide.

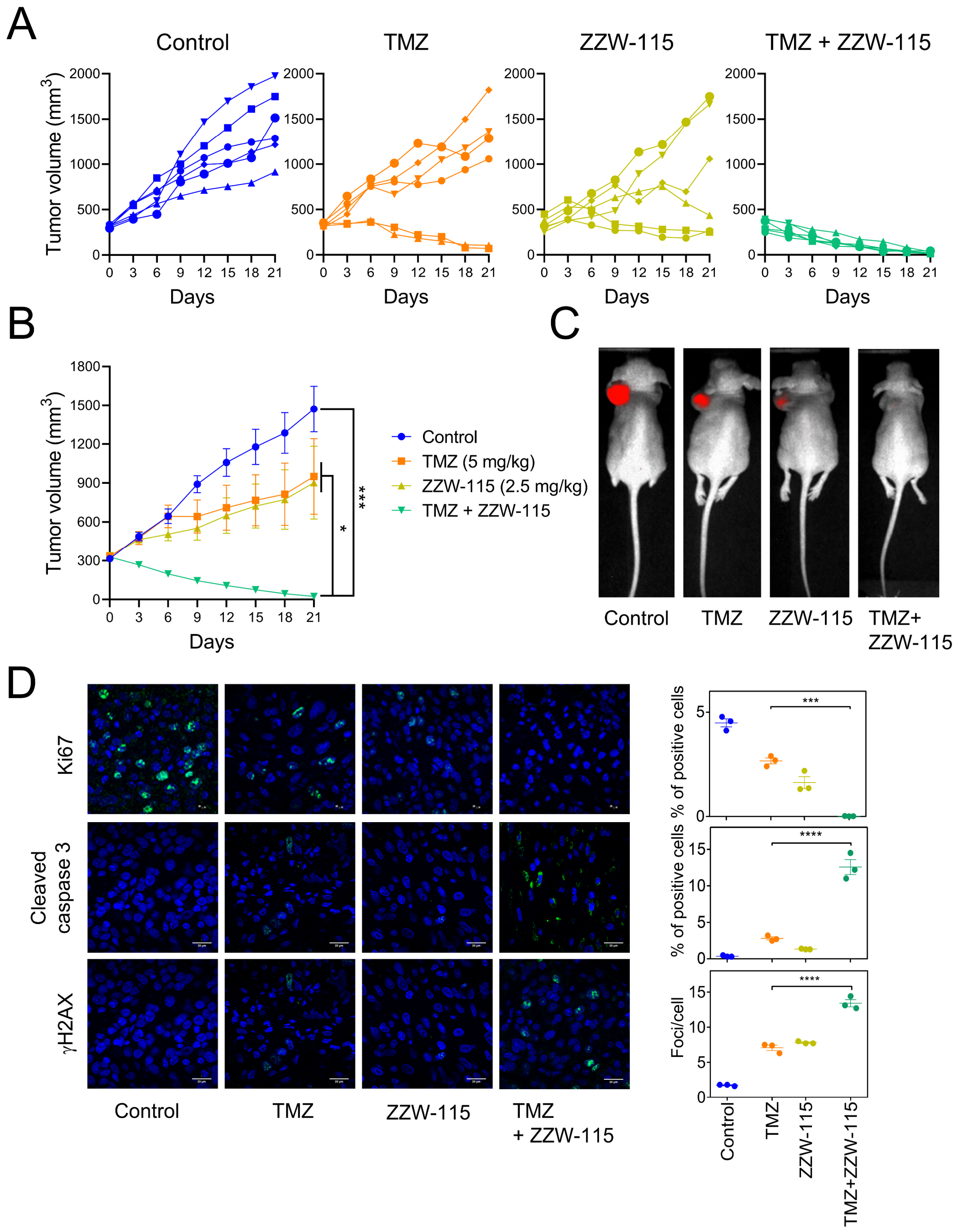

Supplementary Figure 3

ZZW-115 strongly potentiated the anti-tumoral activity on glioblastoma of genotoxic agents *in vivo*. CAnN.Cg-Foxn1nu/Crl BALB/c nude mice xenografted with U87-red cells were separated into 4 groups of 6 mice and treated daily for 21 days with 0.5% DMSO in physiologic serum (control group), 5 mg/kg TMZ, 2.5 mg/kg ZZW-115 or 5 mg/kg TMZ in combination with

2.5 mg/kg ZZW-115. Tumor volume was measured every 3 days. Individual volume of each mouse (A) and mean of the volume of each treatment (B) are shown. For each treatment, statistical significance is *$p < 0.05$ and ***$p < 0.001$ (one-way ANOVA, Tukey's post hoc test). (C) Fluorescent tumors. Pictures of representative animals from each group at the end of the treatments. (D) Immunostaining of tumor samples with antibodies against Ki67, cleaved caspase 3 and γH2AX. Quantification of foci was performed by Image J software on three samples of each group. For each treatment, statistical significance is ***$p < 0.001$ (one-way ANOVA, Tukey's post hoc test). Data represent mean ± SEM, n = 3.

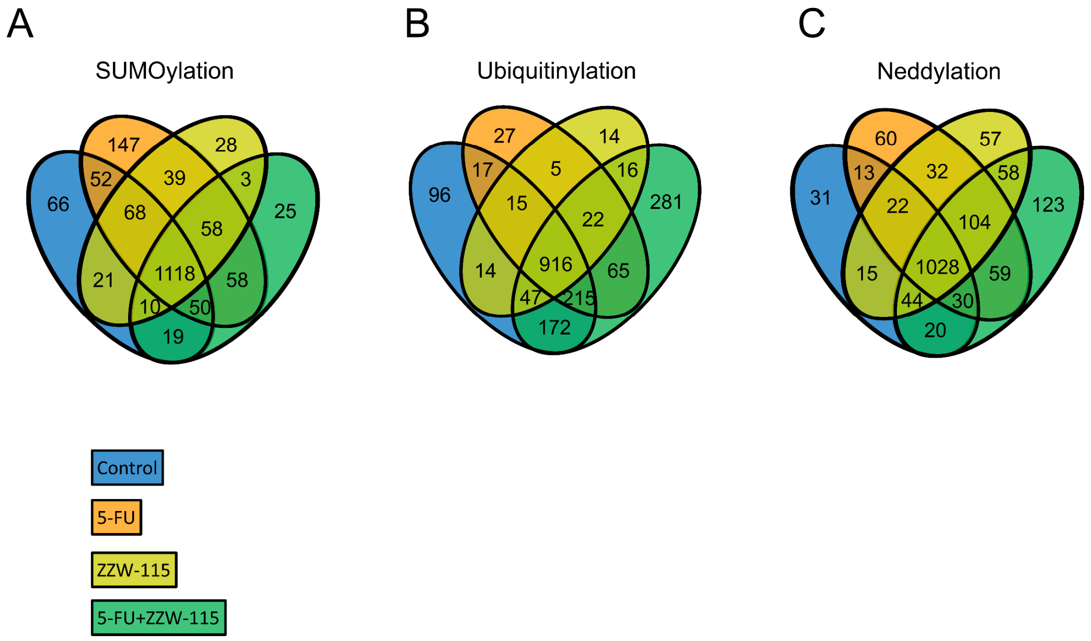

Supplementary Figure 4

Venn Diagram representing the repartition of (A) SUMOylated, (B) Ubiquitylated, and (C) Neddylated proteins among the different conditions (no treatment, 5-FU, ZZW-115, and their combination).

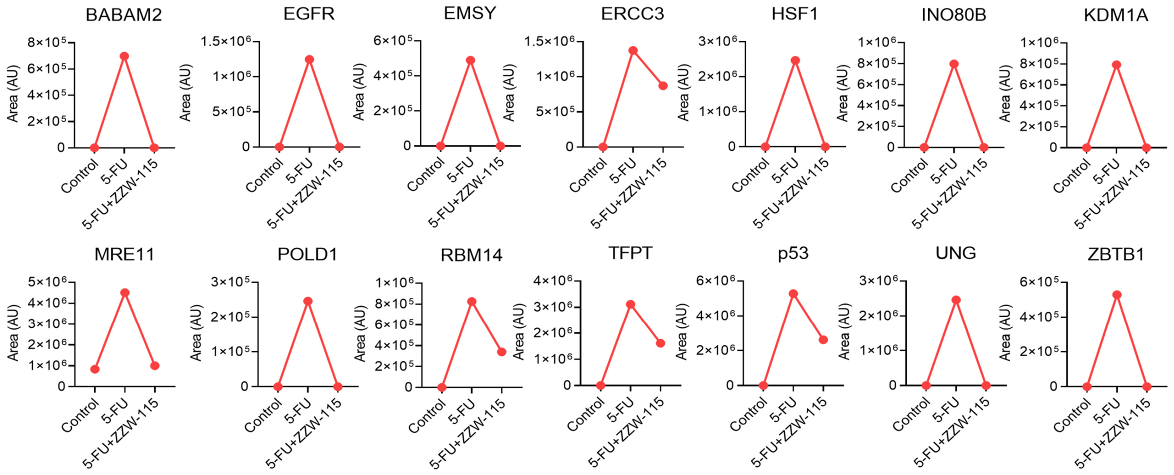

Supplementary Figure 5

Mean of peak area values measurements, in three different conditions (no treatment, 5-FU, and combination of 5-FU with ZZW-115), for the 14 proteins involved in DNA repair and with increased SUMOylation upon 5-FU treatment.
