# Supplementary material for: ZZW-115–dependent inhibition of NUPR1 nuclear translocation sensitizes cancer cells to genotoxic agents
Source: JCI Insight. 2020 Sep 17;5(18):e138117. doi: 10.1172/jci.insight.138117 (PMC7526551; doi:10.1172/jci.insight.138117)
Supplement: Supplemental Table 3 [file jciinsight-5-138117-s194.docx]

**Supplementary Table 3**

List of proteins (total 319) SUMOylated in response to the 5-FU treatment. In black (317), those in which SUMOylation is inhibited by the ZZW-115 treatment. In red (2), those which were insensitive to the ZZW-115 treatment.

| ABCD1 | CIZ1 | FBXW11 | IMMP2L | MYH10 | PPAN | SKIL | TWISTNB |
| --- | --- | --- | --- | --- | --- | --- | --- |
| ABCF1 | CKAP4 | FERMT2 | IMMT | NAA25 | PRKCSH | SLC25A12 | U2SURP |
| ABHD11 | CLK2 | FKBP10 | IMP4 | NAB1 | PRSS1 | SLC25A13 | UBE2I |
| AC118549.1 | CNOT6L | FOSL2 | IMPDH2 | NANS | PSMD12 | SLC2A1 | UBR2 |
| ACACA | COASY | FOXK2 | INO80B | NARS | PSMD5 | SLC39A7 | UNG |
| ACAT1 | COPB1 | FOXP1 | KDM1A | NBR1 | PTBP3 | SOCS6 | UTP6 |
| ACSL1 | CPT2 | FTSJ3 | KIAA1958 | NCAPG2 | PTGR1 | SON | UXT |
| ADI1 | CS | G3BP2 | KNOP1 | NELFB | PTMA | SP3 | WASF1 |
| ADK | CSDE1 | GAA | KRT33A | NFIC | PTPN2 | SPIN4 | WDR76 |
| ADNP | CTBP2 | GALK1 | KRT6A | NHP2 | PUM3 | SQSTM1 | XRN2 |
| AHCYL2 | CTNND1 | GALNS | KRT9 | NIF3L1 | PUSL1 | STK38L | YDJC |
| AHNAK2 | CTR9 | GAN | LANCL2 | NIT1 | RAB32 | SUPT3H | YIF1A |
| AKAP17A | CYB5R3 | GATA2 | LBR | NLK | RAB8A | SUZ12 | YKT6 |
| ALDH16A1 | D2HGDH | GATA4 | LIMK1 | NOC3L | RAD21 | SVIL | ZBTB1 |
| AP4M1 | DCAF10 | GATD3B | LPP | None | RAF1 | TACO1 | ZBTB25 |
| APBA3 | DCAF6 | GMDS | MAGOHB | NOTCH2 | RAI1 | TADA1 | ZC3H14 |
| APOBEC3G | DDX18 | GNAI2 | MAGT1 | NQO2 | RAVER1 | TATDN2 | ZFP90 |
| ARFGEF1 | DDX31 | GNAS | MAP2 | NUMA1 | RBM10 | TBL1XR1 | ZMYM2 |
| ARHGAP5 | DENND5B | GNB2 | MAP3K20 | ODR4 | RBM12B | TCF25 | ZNF146 |
| ARID4B | DGKQ | GPAT3 | MAP4K3 | OPA1 | RBM14 | TCF3 | ZNF16 |
| ASPH | DHX57 | GPATCH2 | MAP4K5 | OTX1 | RCC1L | TCOF1 | ZNF189 |
| ATG2B | DNAJC13 | GPBP1L1 | MAVS | OXA1L | RHEB | TCP1 | ZNF202 |
| BABAM2 | DPM1 | GRN | MBIP | P4HB | RHOT2 | TENT2 | ZNF205 |
| BCAR3 | DPYSL5 | GTPBP3 | MBLAC2 | PAF1 | RNF31 | TFPT | ZNF207 |
| BCLAF1 | EBP | GUSB | MCCC2 | PARP12 | RPL5 | THOC5 | ZNF235 |
| BCLAF3 | EGFR | HAUS5 | MCM4 | PBRM1 | RPP40 | TIMM44 | ZNF324 |
| BRD7 | EIF2B2 | HAUS6 | MCMBP | PDP1 | RPS6KA3 | TJAP1 | ZNF362 |
| BRMS1L | EIF2B5 | HDHD5 | MED1 | PDPR | RPUSD3 | TJP1 | ZNF384 |
| BRPF1 | EIF3F | HEXB | MED16 | PHF10 | RRM2 | TK2 | ZNF391 |
| C17orf97 | EIF4A3 | HIST1H2BJ | MIPEP | PHF21A | RTN4 | TM9SF1 | ZNF397 |
| C5orf22 | EMC3 | HIST2H3A | MLLT10 | PHKA2 | RTTN | TM9SF4 | ZNF398 |
| CALR | EMSY | HK1 | MLPH | PKN1 | S100A6 | TP53 | ZNF460 |
| CAPRIN1 | EPS8L2 | HNRNPLL | MNT | PLK2 | S100A9 | TPP1 | ZNF496 |
| CAV1 | ERCC3 | HNRNPM | MPHOSPH10 | PLPP1 | SAMM50 | TRAF6 | ZNF579 |
| CCDC50 | ERLIN2 | HNRNPUL1 | MRE11 | PNP | SAP18 | TRIM27 | ZNF668 |
| CCNK | ETFDH | HRNR | MRGBP | POC5 | SEC23A | TSC1 | ZNF672 |
| CCSER2 | F2R | HSF1 | MRPS18B | POLD1 | SEC24B | TSN | ZNF768 |
| CCT3 | FADS1 | HSPH1 | MSANTD4 | POLR3D | SEC63 | TSTA3 | ZNF771 |
| CDC40 | FAR1 | IK | MSN | POLR3E | SERPINB4 | TTC17 | ZSCAN26 |
| CENPB | FBRSL1 | ILK | MYDGF | POR | SHC1 | TUBB6 |  |
